# Supplementary figures and images for: Estimation of non-null SNP effect size distributions enables the detection of enriched genes underlying complex traits
Source: PLoS Genet. 2020 Jun 15;16(6):e1008855. doi: 10.1371/journal.pgen.1008855 (PMC7316356; doi:10.1371/journal.pgen.1008855)

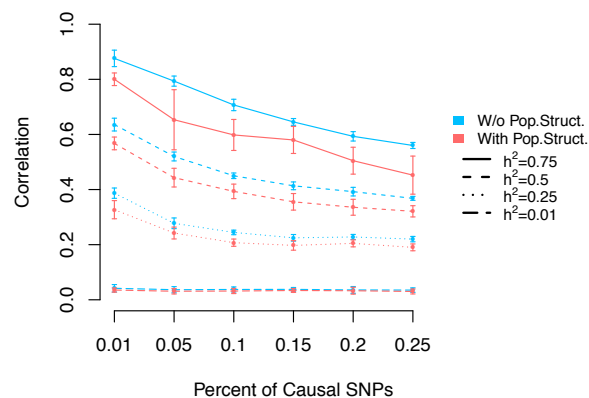

(A) LASSO ( $\alpha = 0$ )

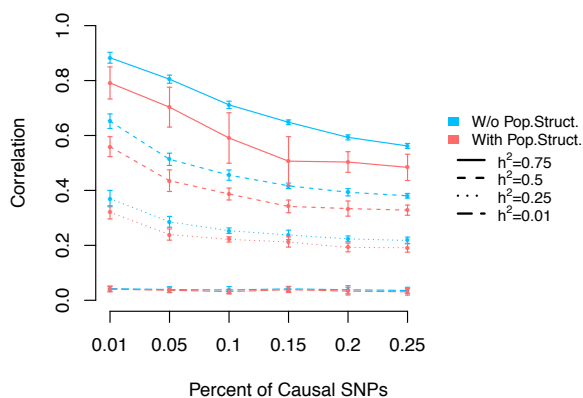

(B) Elastic Net ( $\alpha = 0.5$ )

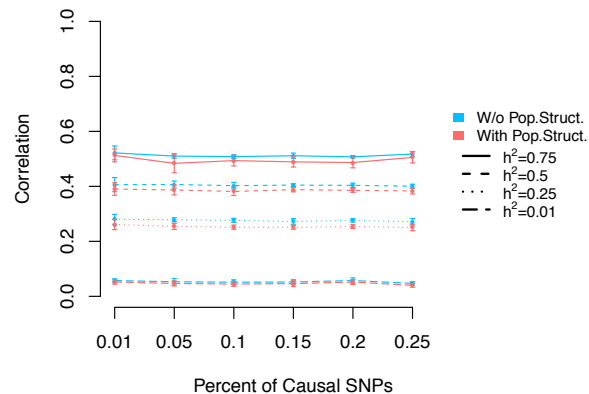

(C) Ridge Regression ( $\alpha = 1$ )

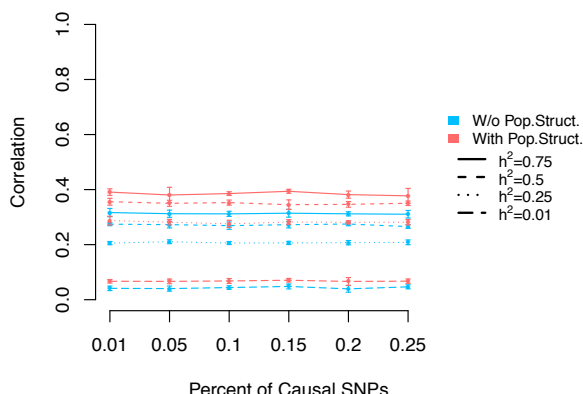

(D) No Regularization

Supplement: S1 Fig — Assessed regularization techniques are the (A) LASSO [23], (B) Elastic Net [24], (C) Ridge Regression [25], and (D) no regularization of ordinary least squares (OLS) effect sizes which serves as a baseline. Here, we take real genotype data on chromosome 19 from N = 5, 000 randomly chosen individuals of European ancestry in the UK Biobank (see S1 Text). We then assumed a simple linear additive model for quantitative traits while varying the narrow-sense heritability (h2 = {0.01, 0.05, 0.10, 0.15, 0.20, 0.25}). We considered two scenarios where traits are generated with and without additional population structure (colored as pink and blue lines, respectively). In the former setting, phenotypes are simulated while also using the top five principal components (PCs) of the genotype matrix as covariates to create stratification. These PCs contributed to 10% of the phenotypic variance. In both settings, GWA SNP-level effect sizes were derived via OLS without accounting for any additional structure. The y-axis shows Pearson correlation between gene-ε regularized effect sizes and the truth. On the x-axis of each plot, we vary the number of causal SNPs for each trait (i.e., {1, 5, 10, 15, 20, 25}%). Results are based on ten replicates (see S1 Text), with the error bars representing standard errors across runs. (PDF) [file pgen.1008855.s001.pdf]

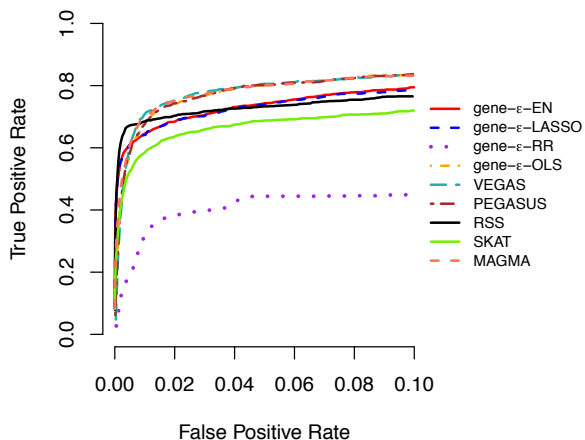

(A) 1% Enriched Genes

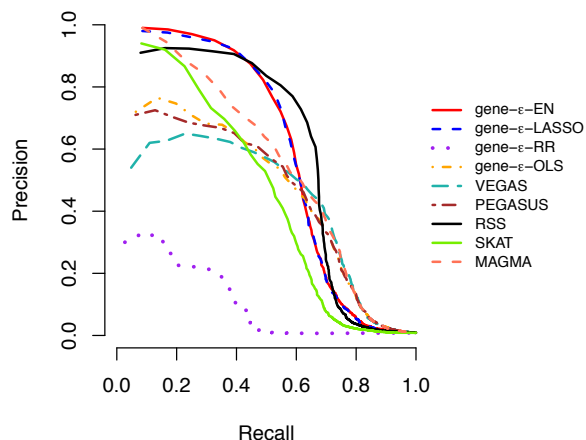

(B) 1% Enriched Genes

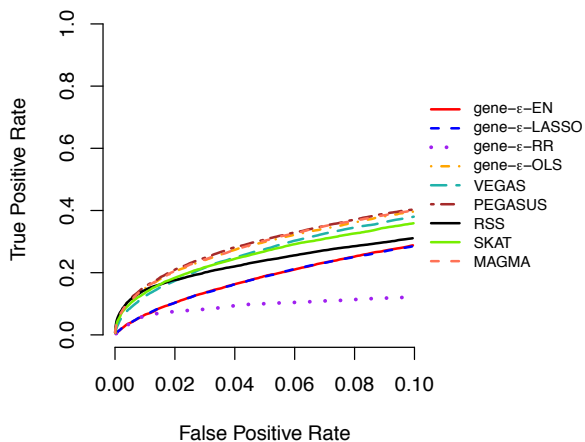

(C) 10% Enriched Genes

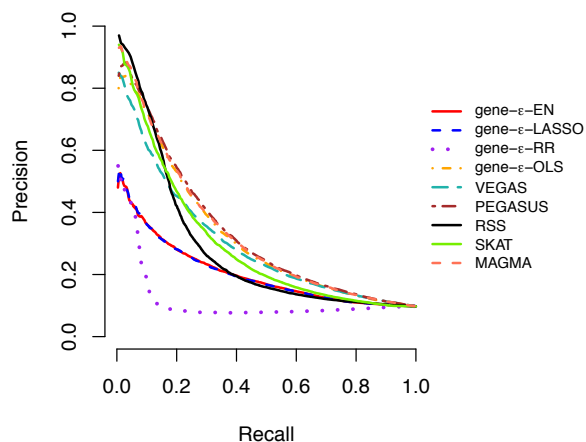

(D) 10% Enriched Genes

Supplement: S2 Fig — Here, the sample size N = 5, 000 and the narrow-sense heritability of the simulated quantitative trait is h2 = 0.2. We compute standard GWA SNP-level effect sizes (estimated using ordinary least squares). Results for gene-ε are shown with LASSO (blue), Elastic Net (EN; red), and Ridge Regression (RR; purple) regularizations. We also show the results of gene-ε without regularization to illustrate the importance of the regularization step (labeled OLS; orange). We compare gene-ε with five existing methods: PEGASUS (brown) [12], VEGAS (teal) [7], the Bayesian approach RSS (black) [14], SKAT (green) [20], and MAGMA (peach) [10]. (A, C) ROC curves show power versus false positive rate for each approach of sparse (1% enriched genes) and polygenic (10% enriched genes) architectures, respectively. Note that the upper limit of the x-axis has been truncated at 0.1. (B, D) Precision-Recall curves for each method applied to the simulations. Note that, in the sparse case (1% enriched genes), the top ranked genes are always true positives, and therefore the minimal recall is not 0. All results are based on 100 replicates (see S1 Text). (PDF) [file pgen.1008855.s002.pdf]

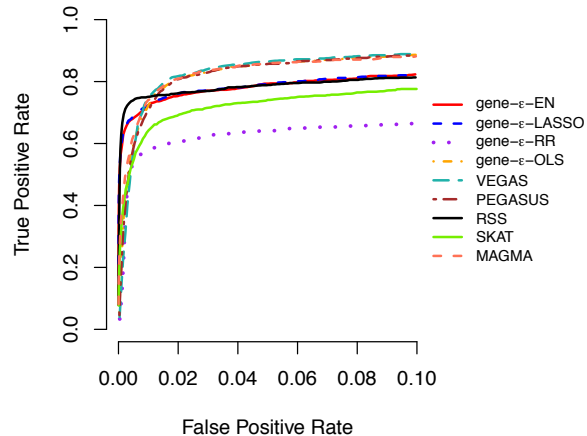

(A) 1% Enriched Genes

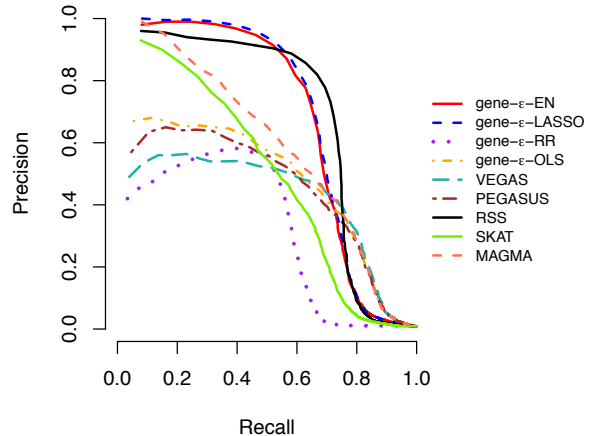

(B) 1% Enriched Genes

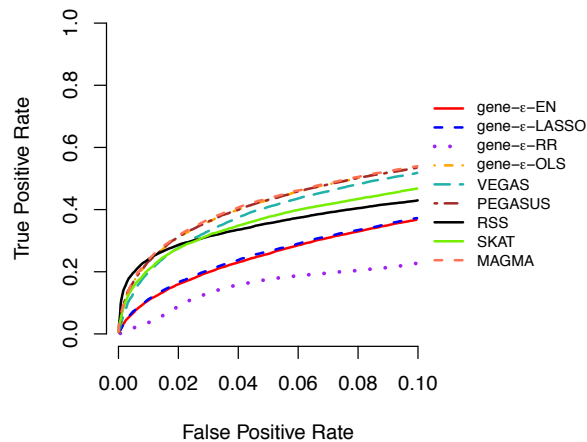

(C) 10% Enriched Genes

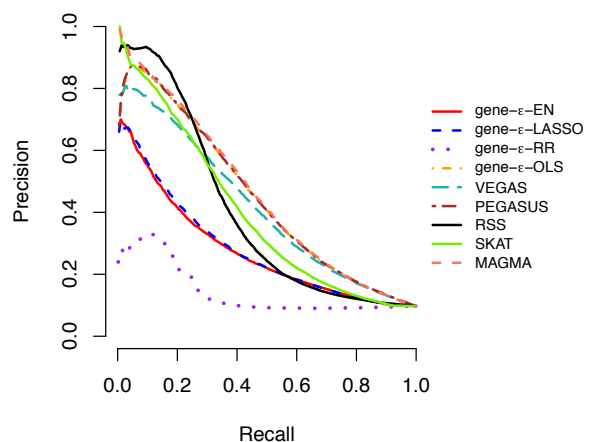

(D) 10% Enriched Genes

Supplement: S3 Fig — Here, the sample size N = 10, 000 and the narrow-sense heritability of the simulated quantitative trait is h2 = 0.2. We compute standard GWA SNP-level effect sizes (estimated using ordinary least squares). Results for gene-ε are shown with LASSO (blue), Elastic Net (EN; red), and Ridge Regression (RR; purple) regularizations. We also show the results of gene-ε without regularization to illustrate the importance of the regularization step (labeled OLS; orange). We compare gene-ε with five existing methods: PEGASUS (brown) [12], VEGAS (teal) [7], the Bayesian approach RSS (black) [14], SKAT (green) [20], and MAGMA (peach) [10]. (A, C) ROC curves show power versus false positive rate for each approach of sparse (1% enriched genes) and polygenic (10% enriched genes) architectures, respectively. Note that the upper limit of the x-axis has been truncated at 0.1. (B, D) Precision-Recall curves for each method applied to the simulations. Note that, in the sparse case (1% enriched genes), the top ranked genes are always true positives, and therefore the minimal recall is not 0. All results are based on 100 replicates (see S1 Text). (PDF) [file pgen.1008855.s003.pdf]

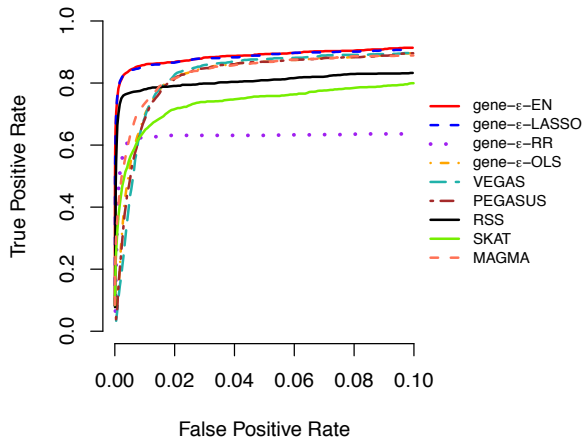

(A) 1% Enriched Genes

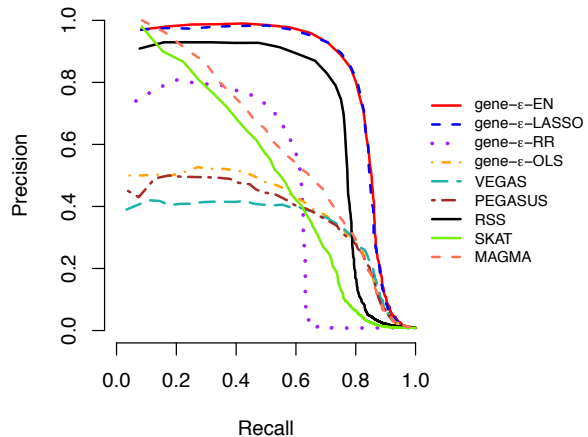

(B) 1% Enriched Genes

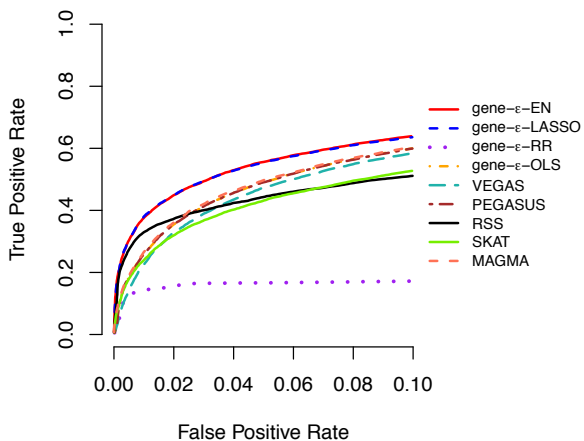

(C) 10% Enriched Genes

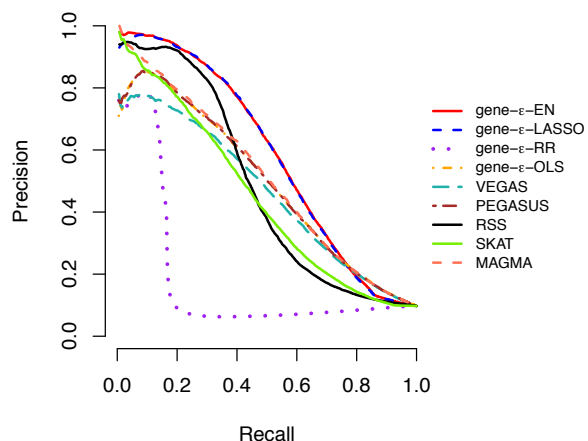

(D) 10% Enriched Genes

Supplement: S4 Fig — Here, the sample size N = 5, 000 and the narrow-sense heritability of the simulated quantitative trait is h2 = 0.6. We compute standard GWA SNP-level effect sizes (estimated using ordinary least squares). Results for gene-ε are shown with LASSO (blue), Elastic Net (EN; red), and Ridge Regression (RR; purple) regularizations. We also show the results of gene-ε without regularization to illustrate the importance of the regularization step (labeled OLS; orange). We compare gene-ε with five existing methods: PEGASUS (brown) [12], VEGAS (teal) [7], the Bayesian approach RSS (black) [14], SKAT (green) [20], and MAGMA (peach) [10]. (A, C) ROC curves show power versus false positive rate for each approach of sparse (1% enriched genes) and polygenic (10% enriched genes) architectures, respectively. Note that the upper limit of the x-axis has been truncated at 0.1. (B, D) Precision-Recall curves for each method applied to the simulations. Note that, in the sparse case (1% enriched genes), the top ranked genes are always true positives, and therefore the minimal recall is not 0. All results are based on 100 replicates (see S1 Text). (PDF) [file pgen.1008855.s004.pdf]

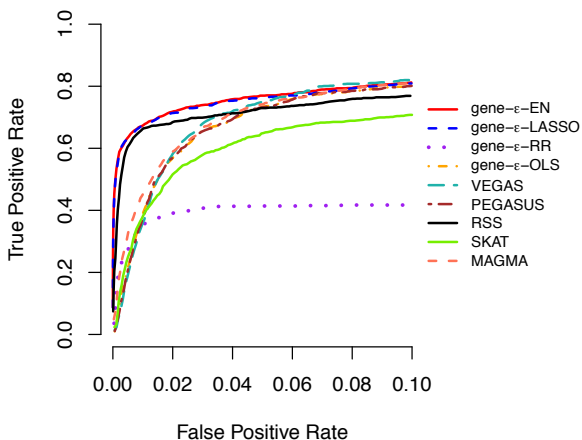

(A) 1% Enriched Genes

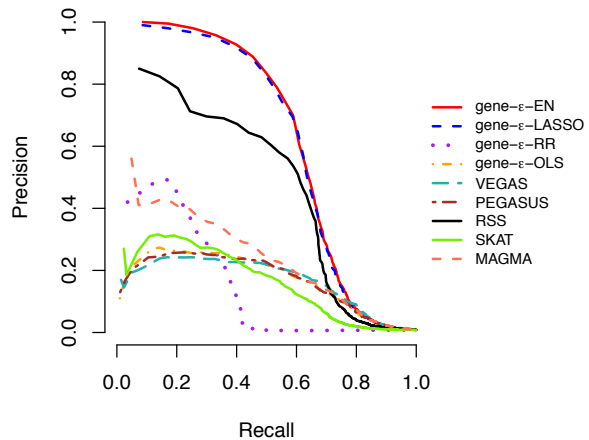

(B) 1% Enriched Genes

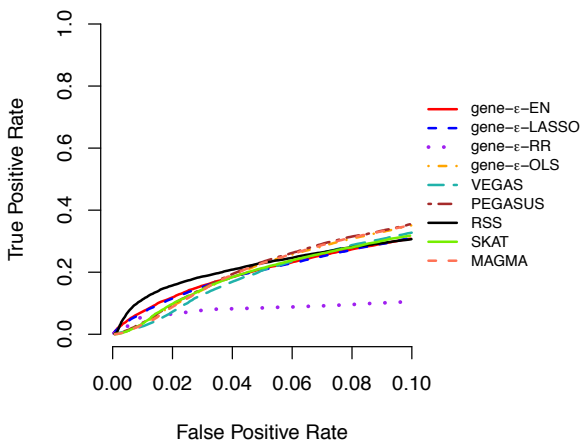

(C) 10% Enriched Genes

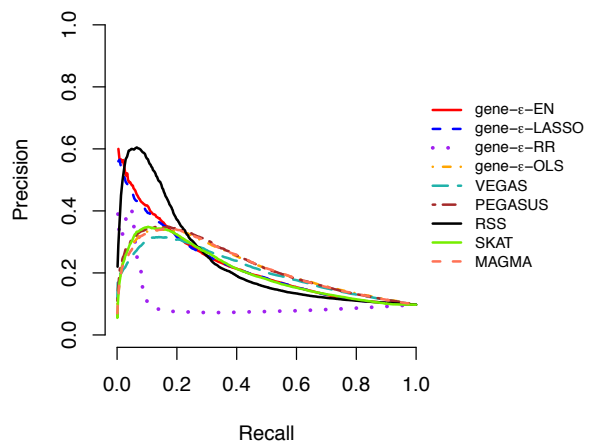

(D) 10% Enriched Genes

Supplement: S5 Fig — Here, the sample size N = 5, 000 and the narrow-sense heritability of the simulated quantitative trait is h2 = 0.2. In this simulation, traits were generated while using the top five principal components (PCs) of the genotype matrix as covariates. GWA summary statistics were computed by fitting a single-SNP univariate linear model (via ordinary least squares) without any control for the additional structure. Results for gene-ε are shown with LASSO (blue), Elastic Net (EN; red), and Ridge Regression (RR; purple) regularizations. We also show the results of gene-ε without regularization to illustrate the importance of the regularization step (labeled OLS; orange). We compare gene-ε with five existing methods: PEGASUS (brown) [12], VEGAS (teal) [7], the Bayesian approach RSS (black) [14], SKAT (green) [20], and MAGMA (peach) [10]. Note that each was method implemented without using any covariates. (A, C) ROC curves show power versus false positive rate for each approach of sparse (1% enriched genes) and polygenic (10% enriched genes) architectures, respectively. Note that the upper limit of the x-axis has been truncated at 0.1. (B, D) Precision-Recall curves for each method applied to the simulations. Note that, in the sparse case (1% enriched genes), the top ranked genes are always true positives, and therefore the minimal recall is not 0. All results are based on 100 replicates (see S1 Text). (PDF) [file pgen.1008855.s005.pdf]

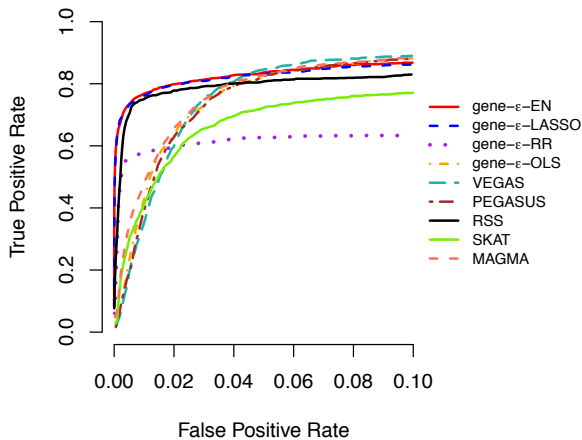

(A) 1% Enriched Genes

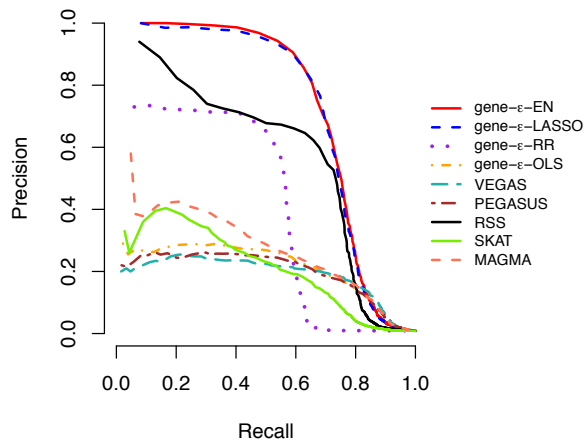

(B) 1% Enriched Genes

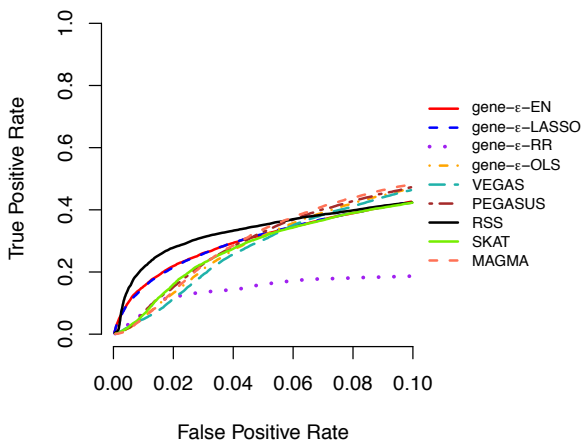

(C) 10% Enriched Genes

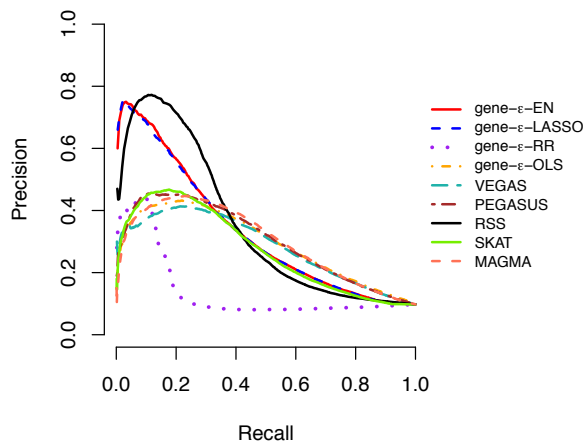

(D) 10% Enriched Genes

Supplement: S6 Fig — Here, the sample size N = 10, 000 and the narrow-sense heritability of the simulated quantitative trait is h2 = 0.2. In this simulation, traits were generated while using the top five principal components (PCs) of the genotype matrix as covariates. GWA summary statistics were computed by fitting a single-SNP univariate linear model (via ordinary least squares) without any control for the additional structure. Results for gene-ε are shown with LASSO (blue), Elastic Net (EN; red), and Ridge Regression (RR; purple) regularizations. We also show the results of gene-ε without regularization to illustrate the importance of the regularization step (labeled OLS; orange). We compare gene-ε with five existing methods: PEGASUS (brown) [12], VEGAS (teal) [7], the Bayesian approach RSS (black) [14], SKAT (green) [20], and MAGMA (peach) [10]. Note that each was method implemented without using any covariates. (A, C) ROC curves show power versus false positive rate for each approach of sparse (1% enriched genes) and polygenic (10% enriched genes) architectures, respectively. Note that the upper limit of the x-axis has been truncated at 0.1. (B, D) Precision-Recall curves for each method applied to the simulations. Note that, in the sparse case (1% enriched genes), the top ranked genes are always true positives, and therefore the minimal recall is not 0. All results are based on 100 replicates (see S1 Text). (PDF) [file pgen.1008855.s006.pdf]

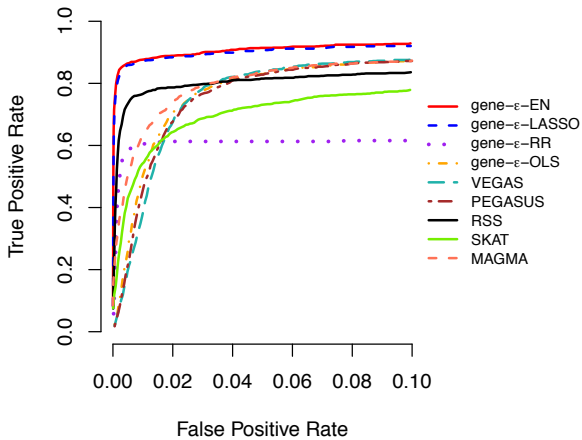

(A) 1% Enriched Genes

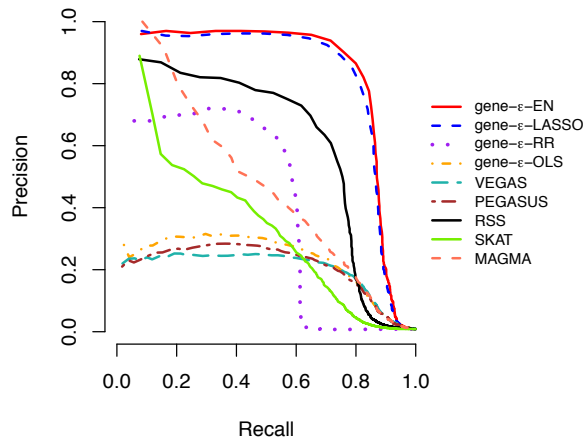

(B) 1% Enriched Genes

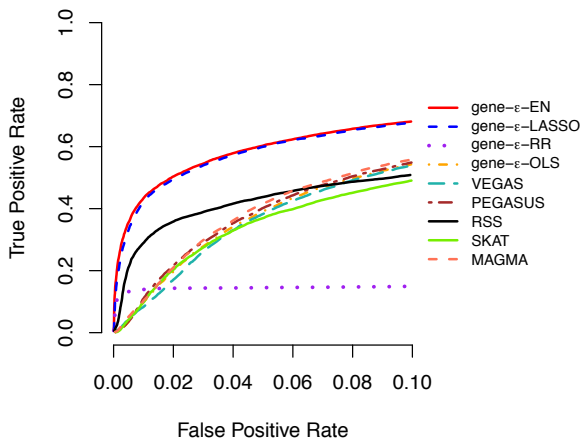

(C) 10% Enriched Genes

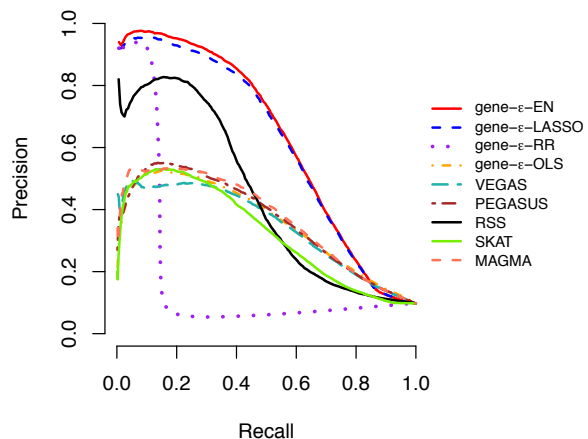

(D) 10% Enriched Genes

Supplement: S7 Fig — Here, the sample size N = 5, 000 and the narrow-sense heritability of the simulated quantitative trait is h2 = 0.6. In this simulation, traits were generated while using the top five principal components (PCs) of the genotype matrix as covariates. GWA summary statistics were computed by fitting a single-SNP univariate linear model (via ordinary least squares) without any control for the additional structure. Results for gene-ε are shown with LASSO (blue), Elastic Net (EN; red), and Ridge Regression (RR; purple) regularizations. We also show the results of gene-ε without regularization to illustrate the importance of the regularization step (labeled OLS; orange). We compare gene-ε with five existing methods: PEGASUS (brown) [12], VEGAS (teal) [7], the Bayesian approach RSS (black) [14], SKAT (green) [20], and MAGMA (peach) [10]. Note that each was method implemented without using any covariates. (A, C) ROC curves show power versus false positive rate for each approach of sparse (1% enriched genes) and polygenic (10% enriched genes) architectures, respectively. Note that the upper limit of the x-axis has been truncated at 0.1. (B, D) Precision-Recall curves for each method applied to the simulations. Note that, in the sparse case (1% enriched genes), the top ranked genes are always true positives, and therefore the minimal recall is not 0. All results are based on 100 replicates (see S1 Text). (PDF) [file pgen.1008855.s007.pdf]

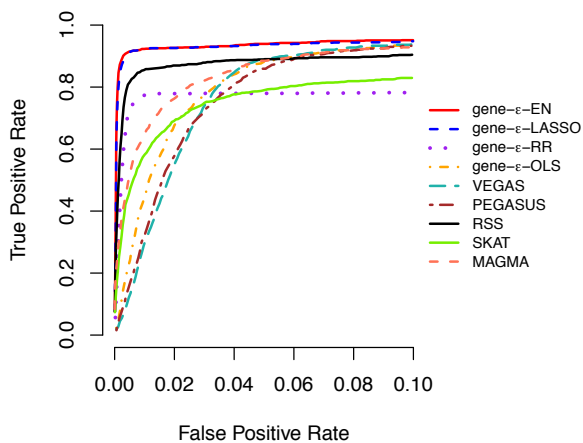

(A) 1% Enriched Genes

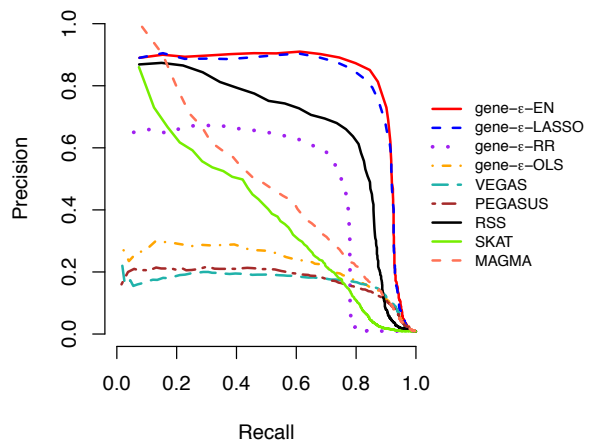

(B) 1% Enriched Genes

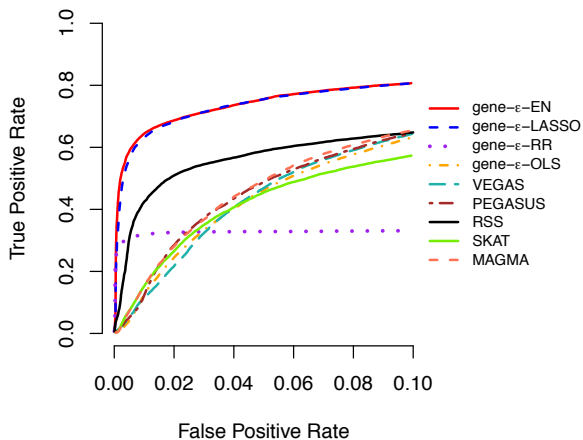

(C) 10% Enriched Genes

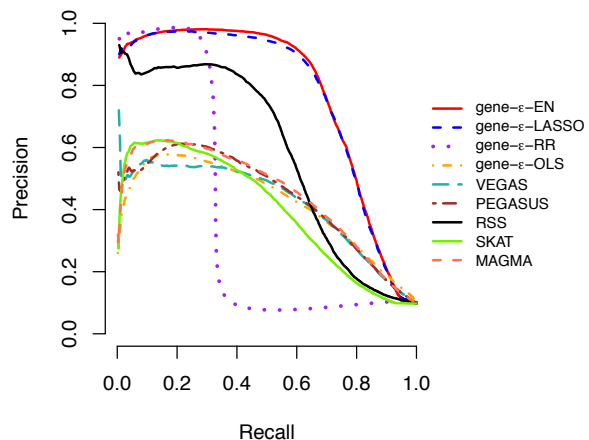

(D) 10% Enriched Genes

Supplement: S8 Fig — Here, the sample size N = 10, 000 and the narrow-sense heritability of the simulated quantitative trait is h2 = 0.6. In this simulation, traits were generated while using the top five principal components (PCs) of the genotype matrix as covariates. GWA summary statistics were computed by fitting a single-SNP univariate linear model (via ordinary least squares) without any control for the additional structure. Results for gene-ε are shown with LASSO (blue), Elastic Net (EN; red), and Ridge Regression (RR; purple) regularizations. We also show the results of gene-ε without regularization to illustrate the importance of the regularization step (labeled OLS; orange). We compare gene-ε with five existing methods: PEGASUS (brown) [12], VEGAS (teal) [7], the Bayesian approach RSS (black) [14], SKAT (green) [20], and MAGMA (peach) [10]. Note that each was method implemented without using any covariates. (A, C) ROC curves show power versus false positive rate for each approach of sparse (1% enriched genes) and polygenic (10% enriched genes) architectures, respectively. Note that the upper limit of the x-axis has been truncated at 0.1. (B, D) Precision-Recall curves for each method applied to the simulations. Note that, in the sparse case (1% enriched genes), the top ranked genes are always true positives, and therefore the minimal recall is not 0. All results are based on 100 replicates (see S1 Text). (PDF) [file pgen.1008855.s008.pdf]

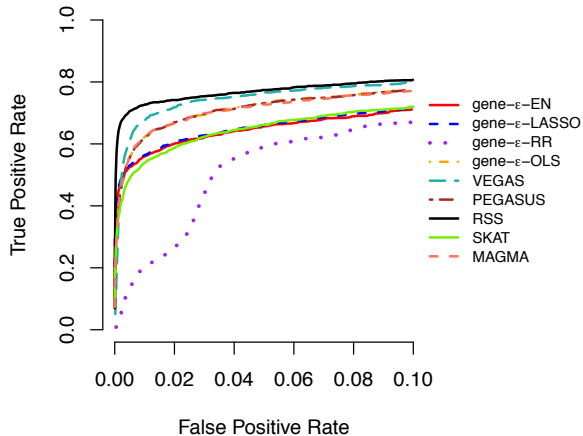

(A) 1% Enriched Genes

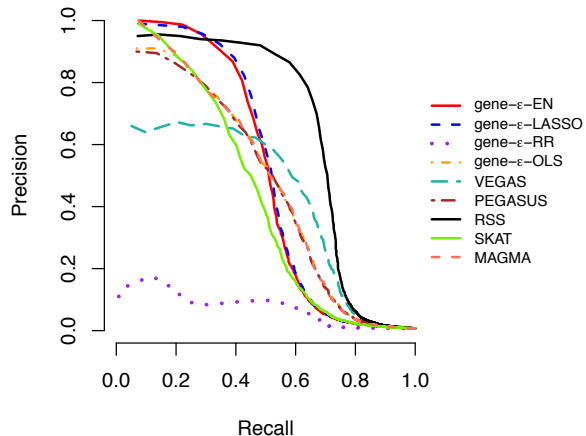

(B) 1% Enriched Genes

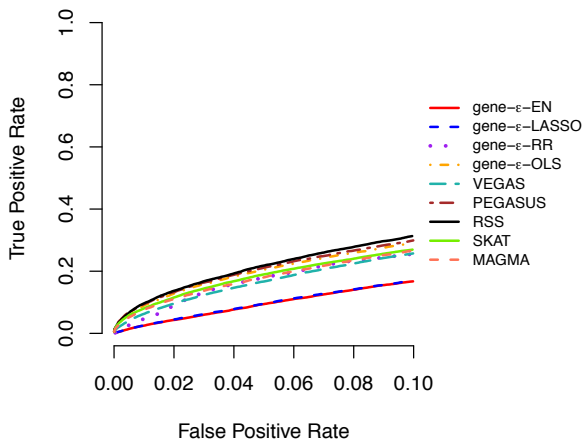

(C) 10% Enriched Genes

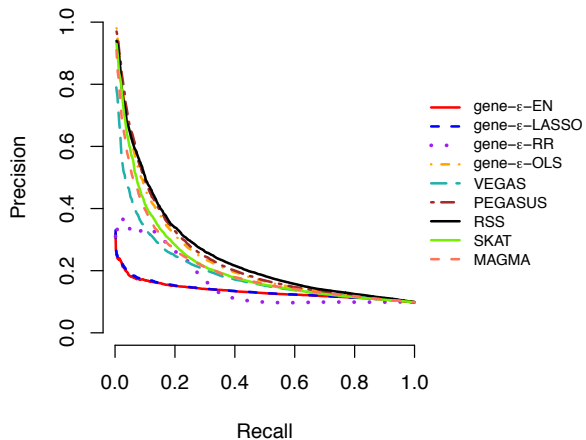

(D) 10% Enriched Genes

Supplement: S9 Fig — Here, the sample size N = 5, 000 and the narrow-sense heritability of the simulated quantitative trait is h2 = 0.2. We compute standard GWA SNP-level effect sizes (estimated using ordinary least squares). Results for gene-ε are shown with LASSO (blue), Elastic Net (EN; red), and Ridge Regression (RR; purple) regularizations. We also show the results of gene-ε without regularization to illustrate the importance of the regularization step (labeled OLS; orange). We compare gene-ε with five existing methods: PEGASUS (brown) [12], VEGAS (teal) [7], the Bayesian approach RSS (black) [14], SKAT (green) [20], and MAGMA (peach) [10]. (A, C) ROC curves show power versus false positive rate for each approach of sparse (1% enriched genes) and polygenic (10% enriched genes) architectures, respectively. Note that the upper limit of the x-axis has been truncated at 0.1. (B, D) Precision-Recall curves for each method applied to the simulations. Note that, in the sparse case (1% enriched genes), the top ranked genes are always true positives, and therefore the minimal recall is not 0. All results are based on 100 replicates (see S1 Text). (PDF) [file pgen.1008855.s009.pdf]

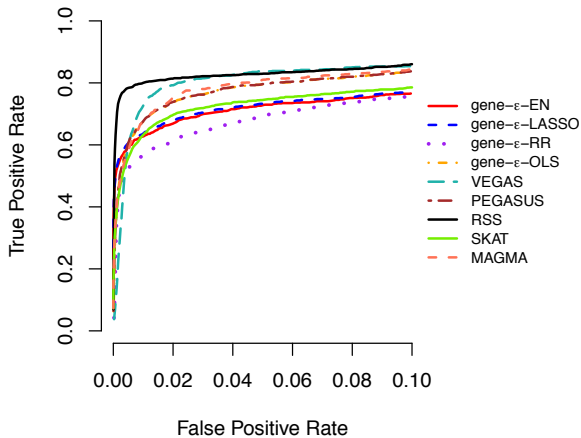

(A) 1% Enriched Genes

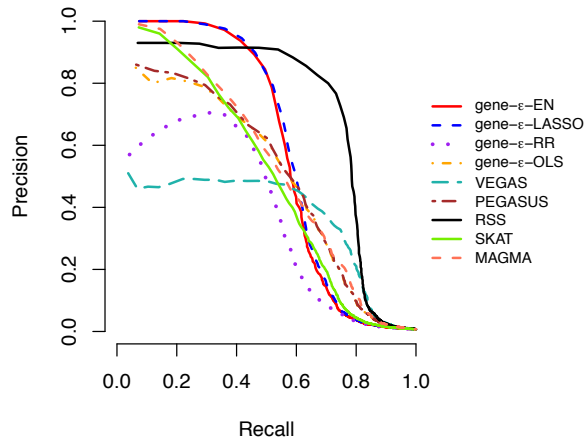

(B) 1% Enriched Genes

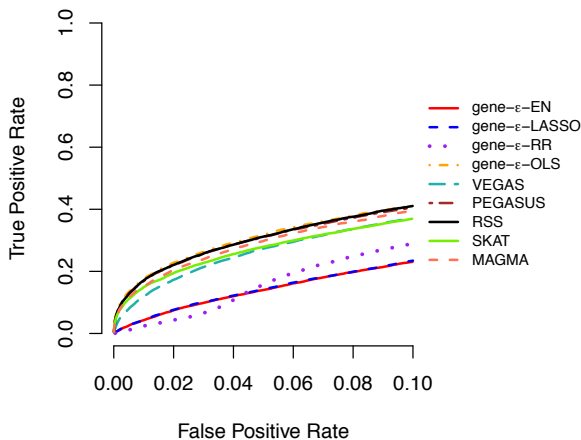

(C) 10% Enriched Genes

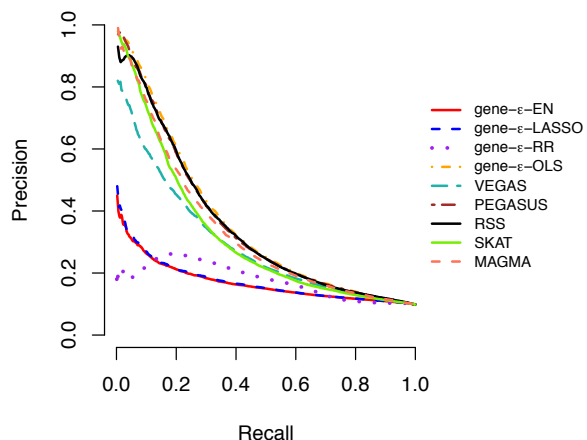

(D) 10% Enriched Genes

Supplement: S10 Fig — Here, the sample size N = 10, 000 and the narrow-sense heritability of the simulated quantitative trait is h2 = 0.2. We compute standard GWA SNP-level effect sizes (estimated using ordinary least squares). Results for gene-ε are shown with LASSO (blue), Elastic Net (EN; red), and Ridge Regression (RR; purple) regularizations. We also show the results of gene-ε without regularization to illustrate the importance of the regularization step (labeled OLS; orange). We compare gene-ε with five existing methods: PEGASUS (brown) [12], VEGAS (teal) [7], the Bayesian approach RSS (black) [14], SKAT (green) [20], and MAGMA (peach) [10]. (A, C) ROC curves show power versus false positive rate for each approach of sparse (1% enriched genes) and polygenic (10% enriched genes) architectures, respectively. Note that the upper limit of the x-axis has been truncated at 0.1. (B, D) Precision-Recall curves for each method applied to the simulations. Note that, in the sparse case (1% enriched genes), the top ranked genes are always true positives, and therefore the minimal recall is not 0. All results are based on 100 replicates (see S1 Text). (PDF) [file pgen.1008855.s010.pdf]

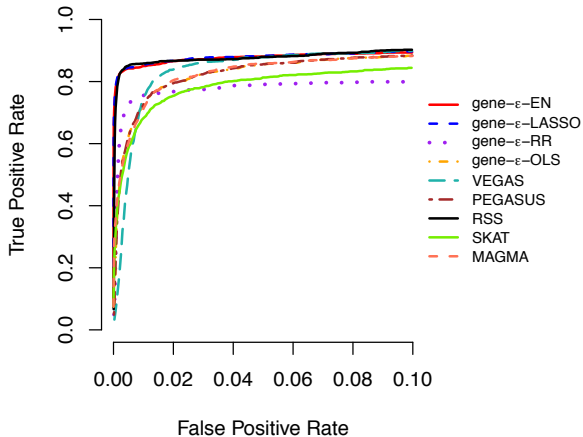

(A) 1% Enriched Genes

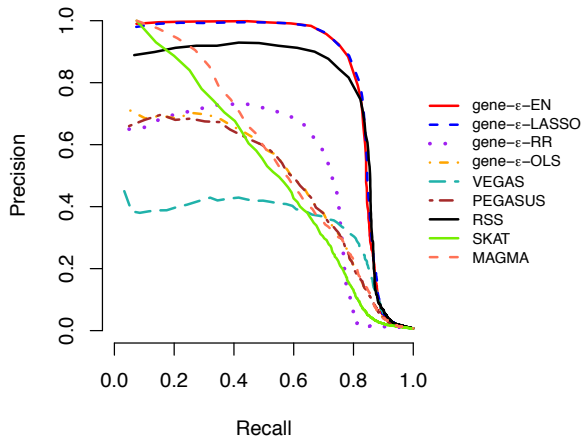

(B) 1% Enriched Genes

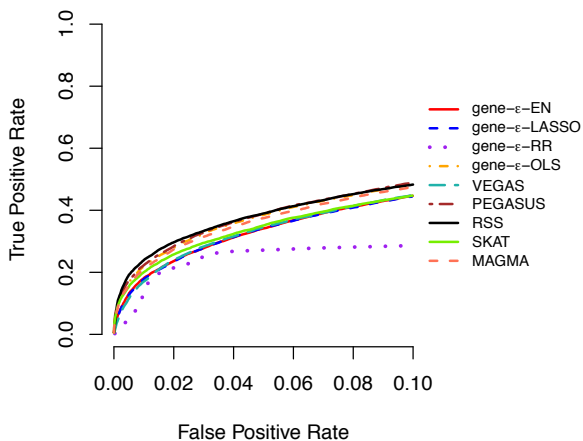

(C) 10% Enriched Genes

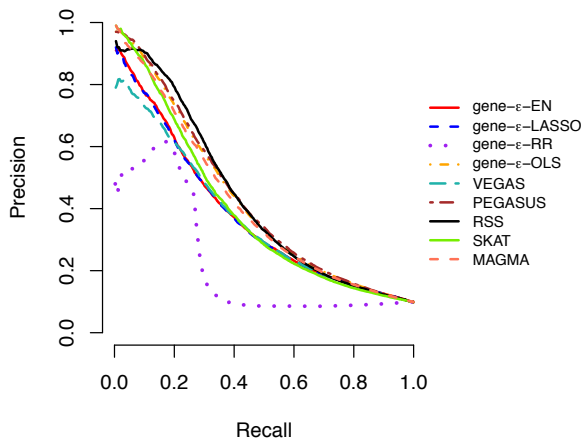

(D) 10% Enriched Genes

Supplement: S11 Fig — Here, the sample size N = 5, 000 and the narrow-sense heritability of the simulated quantitative trait is h2 = 0.6. We compute standard GWA SNP-level effect sizes (estimated using ordinary least squares). Results for gene-ε are shown with LASSO (blue), Elastic Net (EN; red), and Ridge Regression (RR; purple) regularizations. We also show the results of gene-ε without regularization to illustrate the importance of the regularization step (labeled OLS; orange). We compare gene-ε with five existing methods: PEGASUS (brown) [12], VEGAS (teal) [7], the Bayesian approach RSS (black) [14], SKAT (green) [20], and MAGMA (peach) [10]. (A, C) ROC curves show power versus false positive rate for each approach of sparse (1% enriched genes) and polygenic (10% enriched genes) architectures, respectively. Note that the upper limit of the x-axis has been truncated at 0.1. (B, D) Precision-Recall curves for each method applied to the simulations. Note that, in the sparse case (1% enriched genes), the top ranked genes are always true positives, and therefore the minimal recall is not 0. All results are based on 100 replicates (see S1 Text). (PDF) [file pgen.1008855.s011.pdf]

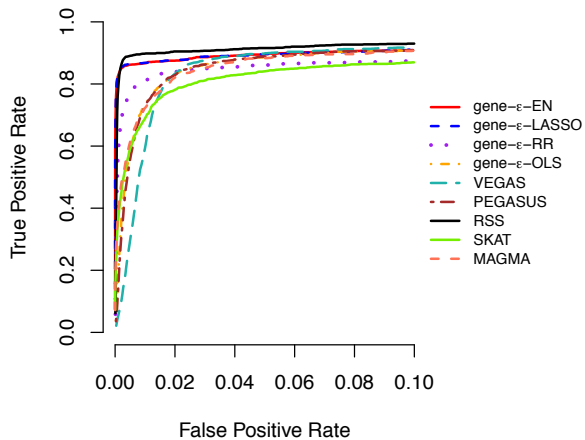

(A) 1% Enriched Genes

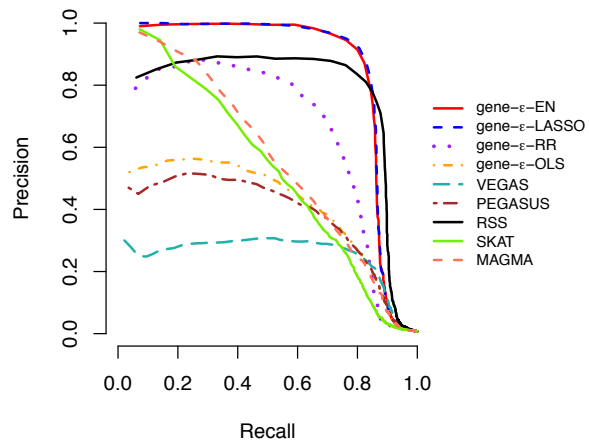

(B) 1% Enriched Genes

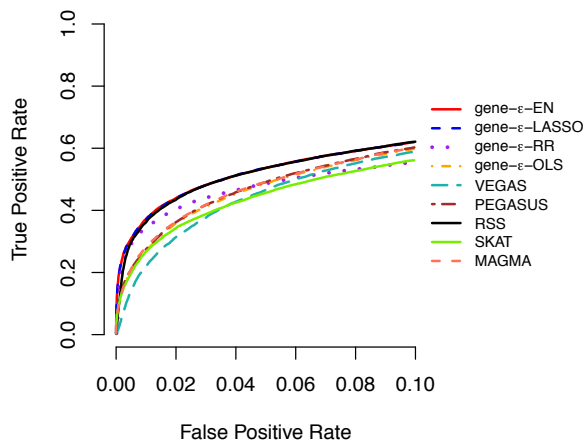

(C) 10% Enriched Genes

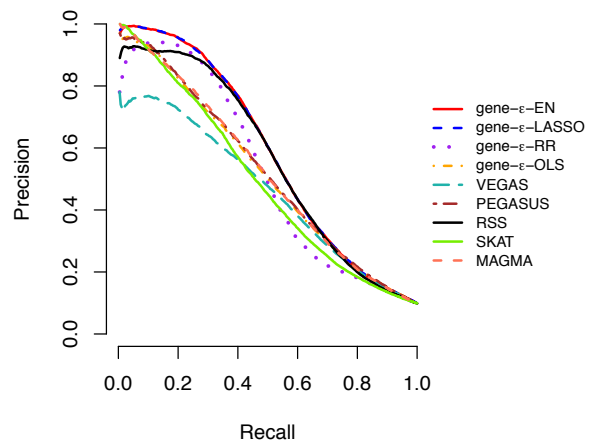

(D) 10% Enriched Genes

Supplement: S12 Fig — Here, the sample size N = 10, 000 and the narrow-sense heritability of the simulated quantitative trait is h2 = 0.6. We compute standard GWA SNP-level effect sizes (estimated using ordinary least squares). Results for gene-ε are shown with LASSO (blue), Elastic Net (EN; red), and Ridge Regression (RR; purple) regularizations. We also show the results of gene-ε without regularization to illustrate the importance of the regularization step (labeled OLS; orange). We compare gene-ε with five existing methods: PEGASUS (brown) [12], VEGAS (teal) [7], the Bayesian approach RSS (black) [14], SKAT (green) [20], and MAGMA (peach) [10]. (A, C) ROC curves show power versus false positive rate for each approach of sparse (1% enriched genes) and polygenic (10% enriched genes) architectures, respectively. Note that the upper limit of the x-axis has been truncated at 0.1. (B, D) Precision-Recall curves for each method applied to the simulations. Note that, in the sparse case (1% enriched genes), the top ranked genes are always true positives, and therefore the minimal recall is not 0. All results are based on 100 replicates (see S1 Text). (PDF) [file pgen.1008855.s012.pdf]

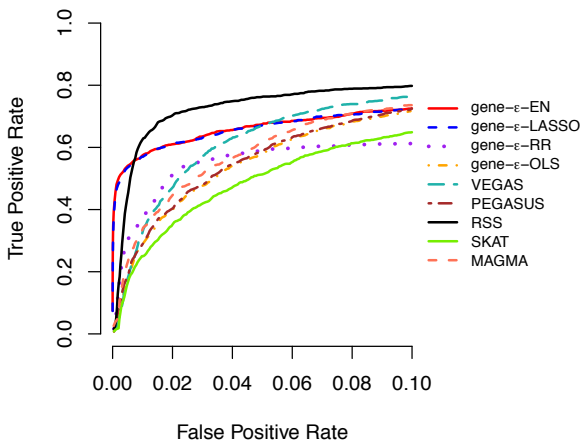

(A) 1% Enriched Genes

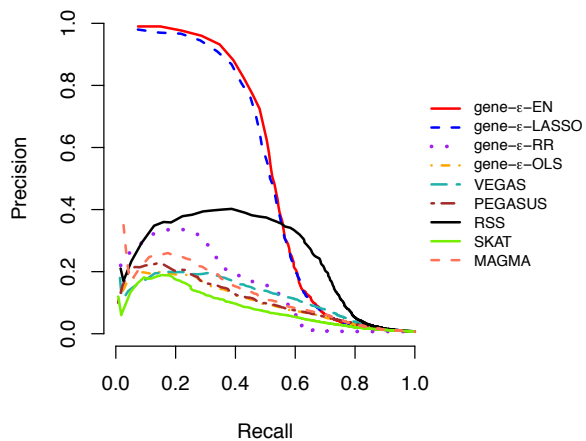

(B) 1% Enriched Genes

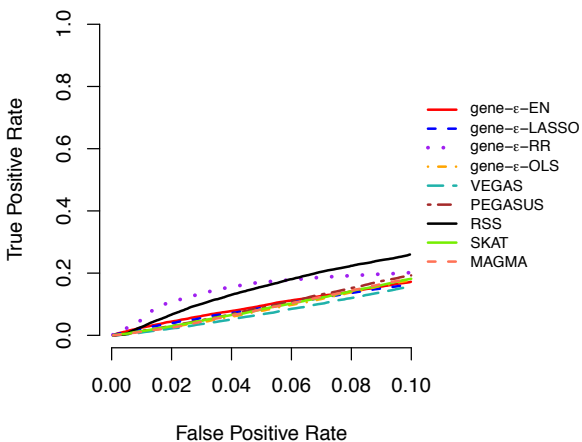

(C) 10% Enriched Genes

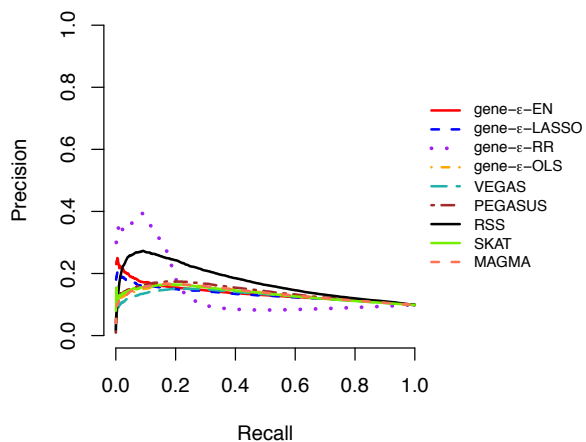

(D) 10% Enriched Genes

Supplement: S13 Fig — Here, the sample size N = 5, 000 and the narrow-sense heritability of the simulated quantitative trait is h2 = 0.2. In this simulation, traits were generated while using the top five principal components (PCs) of the genotype matrix as covariates. GWA summary statistics were computed by fitting a single-SNP univariate linear model (via ordinary least squares) without any control for the additional structure. Results for gene-ε are shown with LASSO (blue), Elastic Net (EN; red), and Ridge Regression (RR; purple) regularizations. We also show the results of gene-ε without regularization to illustrate the importance of the regularization step (labeled OLS; orange). We compare gene-ε with five existing methods: PEGASUS (brown) [12], VEGAS (teal) [7], the Bayesian approach RSS (black) [14], SKAT (green) [20], and MAGMA (peach) [10]. Note that each was method implemented without using any covariates. (A, C) ROC curves show power versus false positive rate for each approach of sparse (1% enriched genes) and polygenic (10% enriched genes) architectures, respectively. Note that the upper limit of the x-axis has been truncated at 0.1. (B, D) Precision-Recall curves for each method applied to the simulations. Note that, in the sparse case (1% enriched genes), the top ranked genes are always true positives, and therefore the minimal recall is not 0. All results are based on 100 replicates (see S1 Text). (PDF) [file pgen.1008855.s013.pdf]

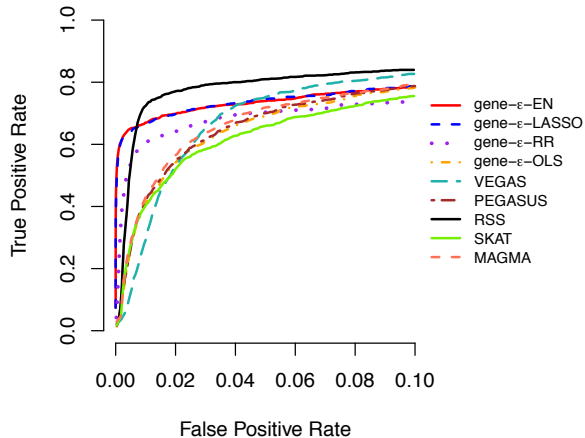

(A) 1% Enriched Genes

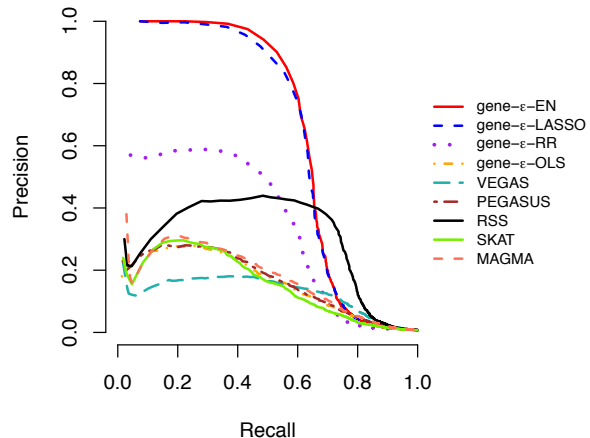

(B) 1% Enriched Genes

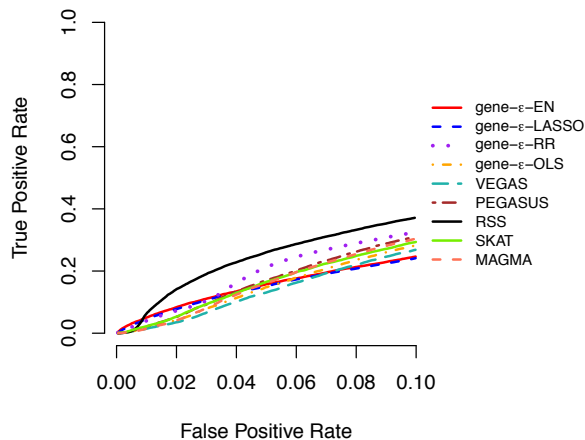

(C) 10% Enriched Genes

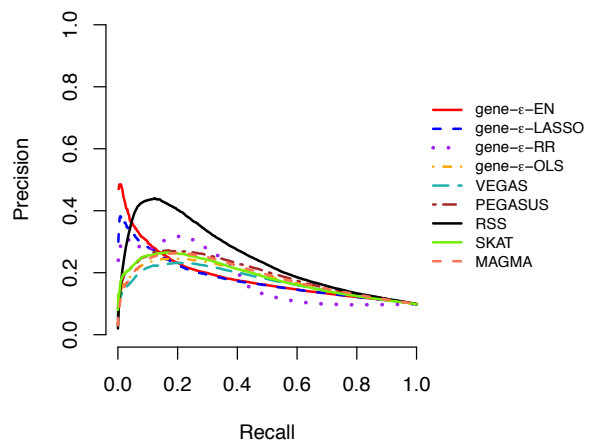

(D) 10% Enriched Genes

Supplement: S14 Fig — Here, the sample size N = 10, 000 and the narrow-sense heritability of the simulated quantitative trait is h2 = 0.2. In this simulation, traits were generated while using the top five principal components (PCs) of the genotype matrix as covariates. GWA summary statistics were computed by fitting a single-SNP univariate linear model (via ordinary least squares) without any control for the additional structure. Results for gene-ε are shown with LASSO (blue), Elastic Net (EN; red), and Ridge Regression (RR; purple) regularizations. We also show the results of gene-ε without regularization to illustrate the importance of the regularization step (labeled OLS; orange). We compare gene-ε with five existing methods: PEGASUS (brown) [12], VEGAS (teal) [7], the Bayesian approach RSS (black) [14], SKAT (green) [20], and MAGMA (peach) [10]. Note that each was method implemented without using any covariates. (A, C) ROC curves show power versus false positive rate for each approach of sparse (1% enriched genes) and polygenic (10% enriched genes) architectures, respectively. Note that the upper limit of the x-axis has been truncated at 0.1. (B, D) Precision-Recall curves for each method applied to the simulations. Note that, in the sparse case (1% enriched genes), the top ranked genes are always true positives, and therefore the minimal recall is not 0. All results are based on 100 replicates (see S1 Text). (PDF) [file pgen.1008855.s014.pdf]

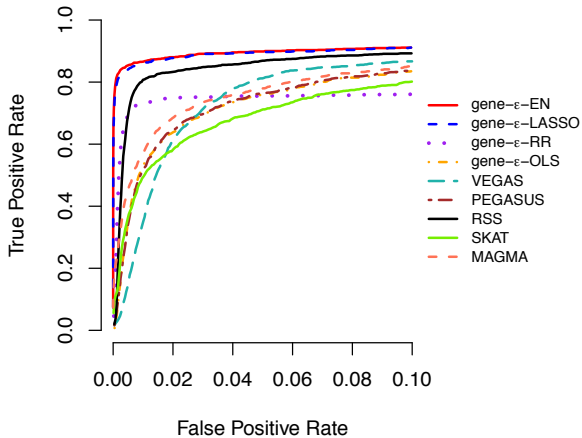

(A) 1% Enriched Genes

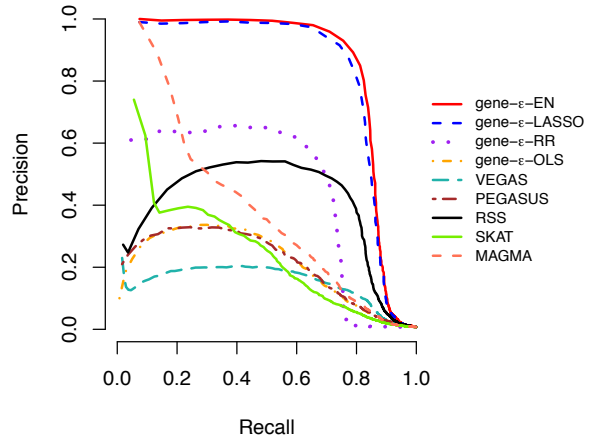

(B) 1% Enriched Genes

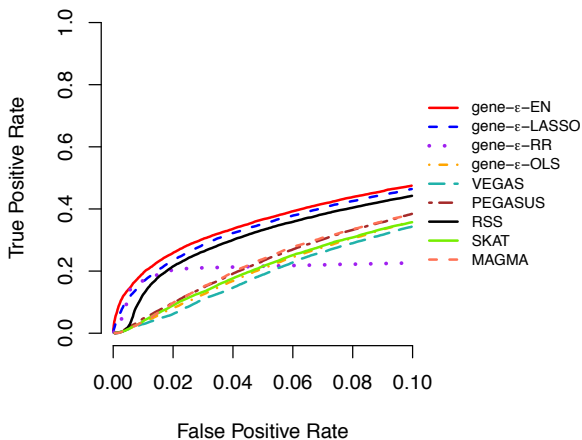

(C) 10% Enriched Genes

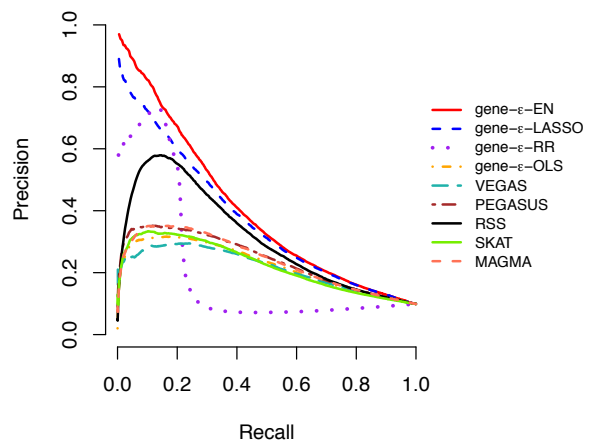

(D) 10% Enriched Genes

Supplement: S15 Fig — Here, the sample size N = 5, 000 and the narrow-sense heritability of the simulated quantitative trait is h2 = 0.6. In this simulation, traits were generated while using the top five principal components (PCs) of the genotype matrix as covariates. GWA summary statistics were computed by fitting a single-SNP univariate linear model (via ordinary least squares) without any control for the additional structure. Results for gene-ε are shown with LASSO (blue), Elastic Net (EN; red), and Ridge Regression (RR; purple) regularizations. We also show the results of gene-ε without regularization to illustrate the importance of the regularization step (labeled OLS; orange). We compare gene-ε with five existing methods: PEGASUS (brown) [12], VEGAS (teal) [7], the Bayesian approach RSS (black) [14], SKAT (green) [20], and MAGMA (peach) [10]. Note that each was method implemented without using any covariates. (A, C) ROC curves show power versus false positive rate for each approach of sparse (1% enriched genes) and polygenic (10% enriched genes) architectures, respectively. Note that the upper limit of the x-axis has been truncated at 0.1. (B, D) Precision-Recall curves for each method applied to the simulations. Note that, in the sparse case (1% enriched genes), the top ranked genes are always true positives, and therefore the minimal recall is not 0. All results are based on 100 replicates (see S1 Text). (PDF) [file pgen.1008855.s015.pdf]

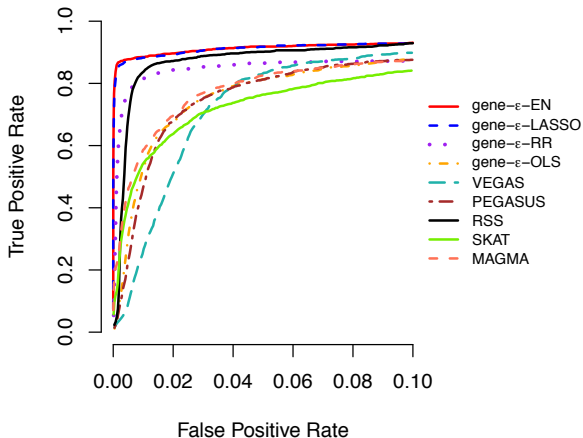

(A) 1% Enriched Genes

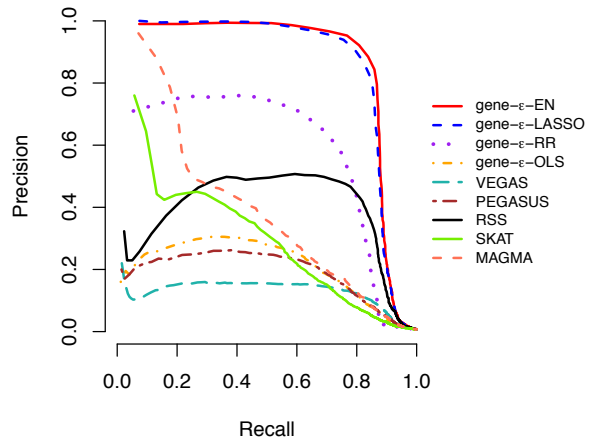

(B) 1% Enriched Genes

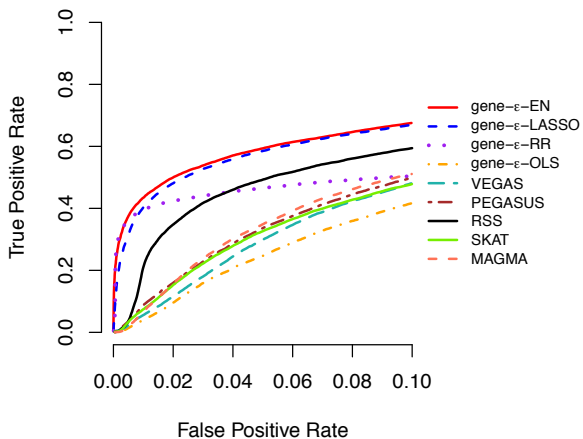

(C) 10% Enriched Genes

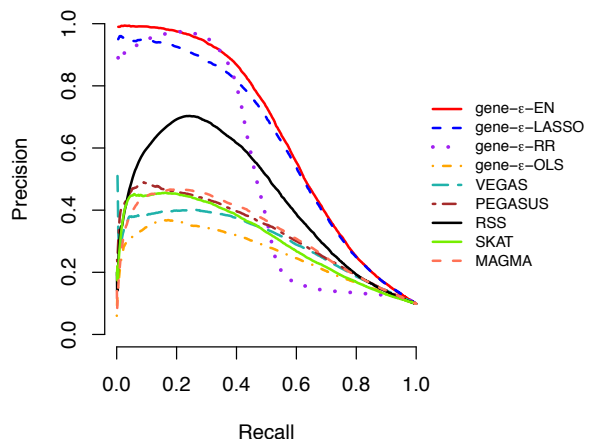

(D) 10% Enriched Genes

Supplement: S16 Fig — Here, the sample size N = 10, 000 and the narrow-sense heritability of the simulated quantitative trait is h2 = 0.6. In this simulation, traits were generated while using the top five principal components (PCs) of the genotype matrix as covariates. GWA summary statistics were computed by fitting a single-SNP univariate linear model (via ordinary least squares) without any control for the additional structure. Results for gene-ε are shown with LASSO (blue), Elastic Net (EN; red), and Ridge Regression (RR; purple) regularizations. We also show the results of gene-ε without regularization to illustrate the importance of the regularization step (labeled OLS; orange). We compare gene-ε with five existing methods: PEGASUS (brown) [12], VEGAS (teal) [7], the Bayesian approach RSS (black) [14], SKAT (green) [20], and MAGMA (peach) [10]. Note that each was method implemented without using any covariates. (A, C) ROC curves show power versus false positive rate for each approach of sparse (1% enriched genes) and polygenic (10% enriched genes) architectures, respectively. Note that the upper limit of the x-axis has been truncated at 0.1. (B, D) Precision-Recall curves for each method applied to the simulations. Note that, in the sparse case (1% enriched genes), the top ranked genes are always true positives, and therefore the minimal recall is not 0. All results are based on 100 replicates (see S1 Text). (PDF) [file pgen.1008855.s016.pdf]

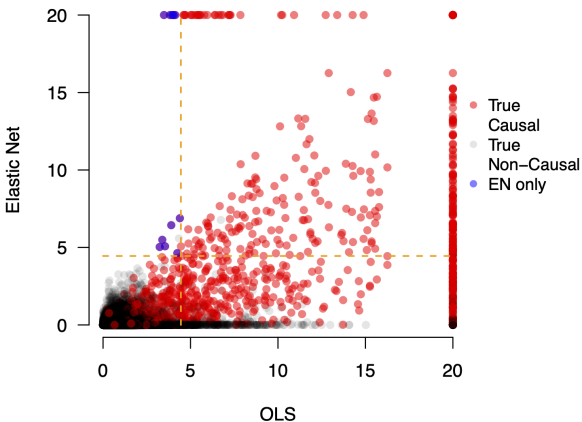

(A)  $N = 5,000$  with 1% Enriched Genes

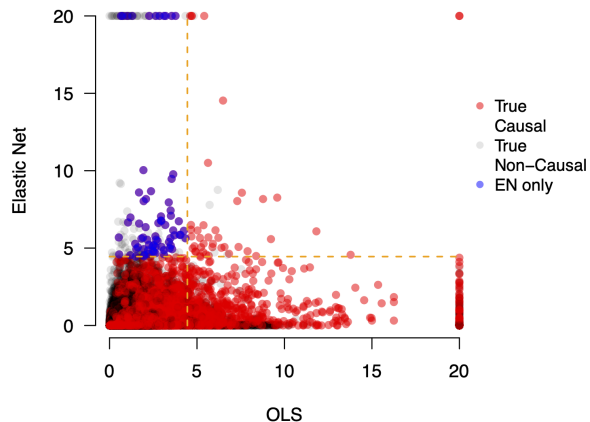

(B)  $N = 5,000$  with 10% Enriched Genes

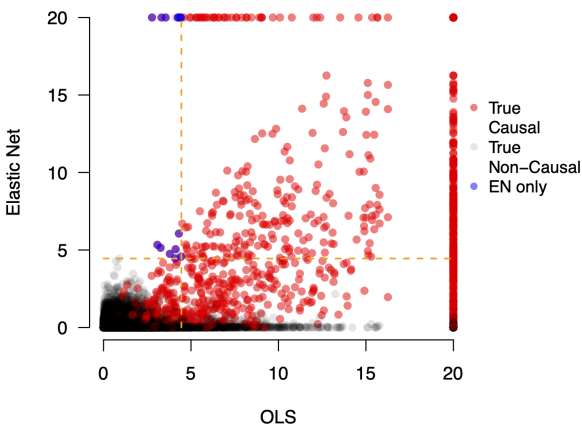

(C)  $N = 10,000$  with 1% Enriched Genes

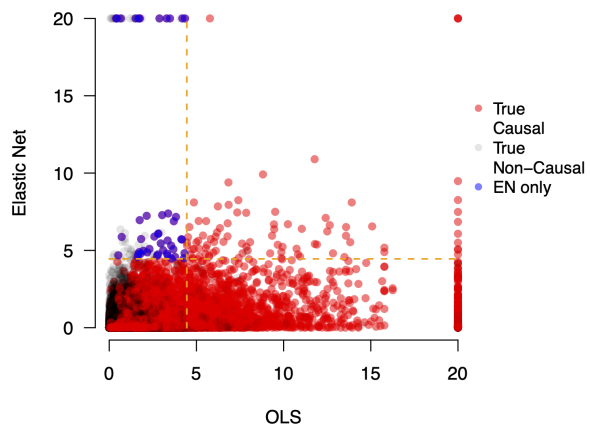

(D)  $N = 10,000$  with 10% Enriched Genes

Supplement: S17 Fig — Here, the narrow-sense heritability of the simulated quantitative traits is h2 = 0.2 and sample sizes are set to N = 5,000 in (A, B) and N = 10,000 in (C, D). In each case, standard GWA summary statistics were computed by fitting a single-SNP univariate linear model (via ordinary least squares). Results are shown comparing the -log10 transformed gene-level P-values derived by gene-ε with Elastic Net (EN) regularization on the y-axis and without regularization (labeled as OLS) on the x-axis. The horizontal and vertical dashed lines are marked at the Bonferonni-corrected threshold P = 3.55×10^−5^ corrected for the 1,408 genes on chromosome 1 from the UK Biobank genotype data. True positive causal genes used to generate the synthetic phenotypes are colored in red, while non-causal genes are given in grey. Genes in the top right quadrant are selected by both approaches. Genes in the top left and bottom right quadrants are uniquely identified by gene-ε-EN and gene-ε-OLS, respectively. To illustrate the importance of regularization on SNP-level summary statistics, we highlight the true positive genes only identified by gene-ε-EN in blue. Each plot combines results from 100 simulated replicates (see S1 Text). (PDF) [file pgen.1008855.s017.pdf]

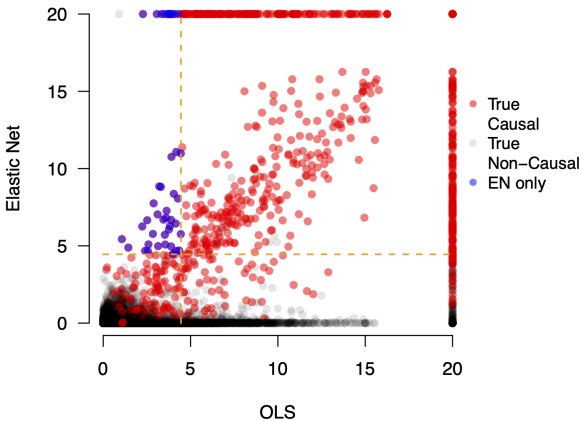

(A)  $N = 5,000$  with 1% Enriched Genes

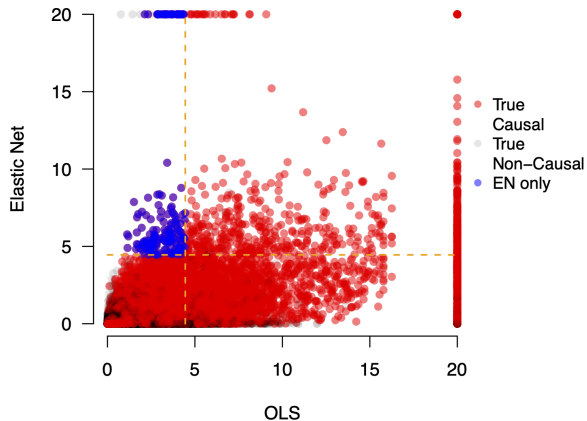

(B)  $N = 5,000$  with 10% Enriched Genes

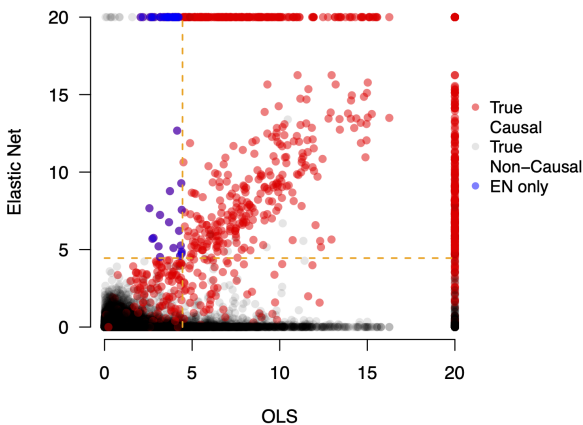

(C)  $N = 10,000$  with 1% Enriched Genes

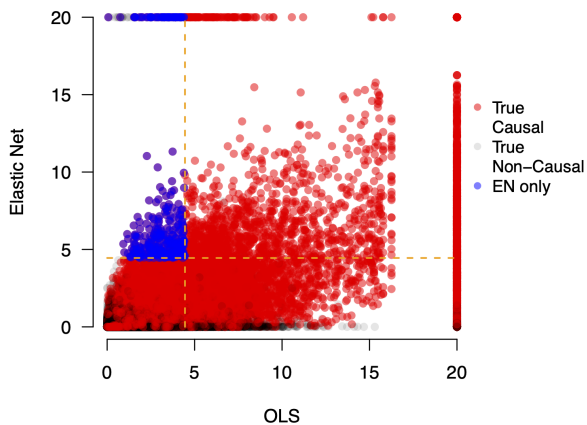

(D)  $N = 10,000$  with 10% Enriched Genes

Supplement: S18 Fig — Here, the narrow-sense heritability of the simulated quantitative traits is h2 = 0.6 and sample sizes are set to N = 5,000 in (A, B) and N = 10,000 in (C, D). In each case, standard GWA summary statistics were computed by fitting a single-SNP univariate linear model (via ordinary least squares). Results are shown comparing the -log10 transformed gene-level P-values derived by gene-ε with Elastic Net (EN) regularization on the y-axis and without regularization (labeled as OLS) on the x-axis. The horizontal and vertical dashed lines are marked at the Bonferonni-corrected threshold Pp = 3.55×10^−5^ corrected for the 1,408 genes on chromosome 1 from the UK Biobank genotype data. True positive causal genes used to generate the synthetic phenotypes are colored in red, while non-causal genes are given in grey. Genes in the top right quadrant are selected by both approaches. Genes in the top left and bottom right quadrants are uniquely identified by gene-ε-EN and gene-ε-OLS, respectively. To illustrate the importance of regularization on SNP-level summary statistics, we highlight the true positive genes only identified by gene-ε-EN in blue. Each plot combines results from 100 simulated replicates (see S1 Text). (PDF) [file pgen.1008855.s018.pdf]

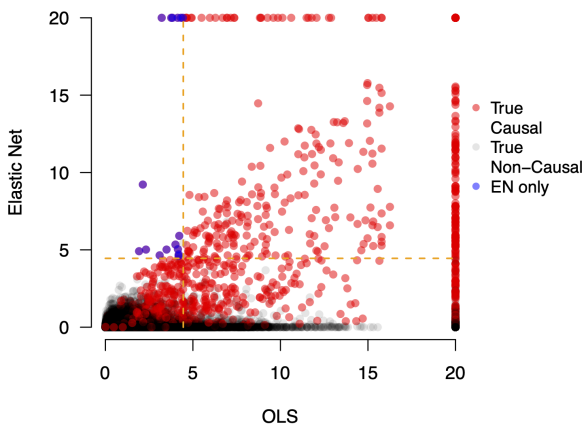

(A)  $N = 5,000$  with 1% Enriched Genes

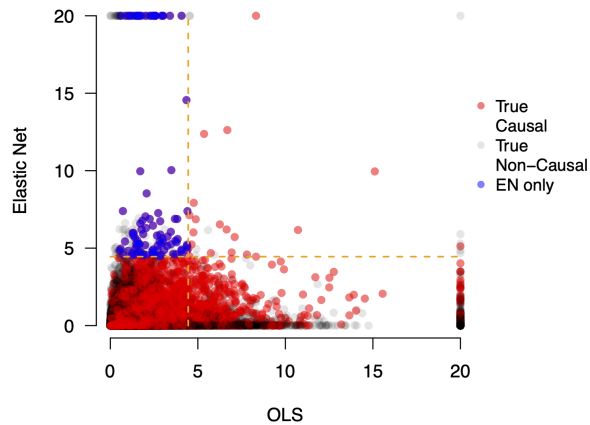

(B)  $N = 5,000$  with 10% Enriched Genes

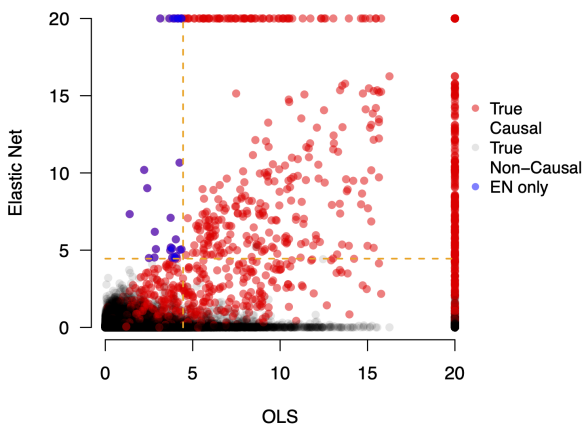

(C)  $N = 10,000$  with 1% Enriched Genes

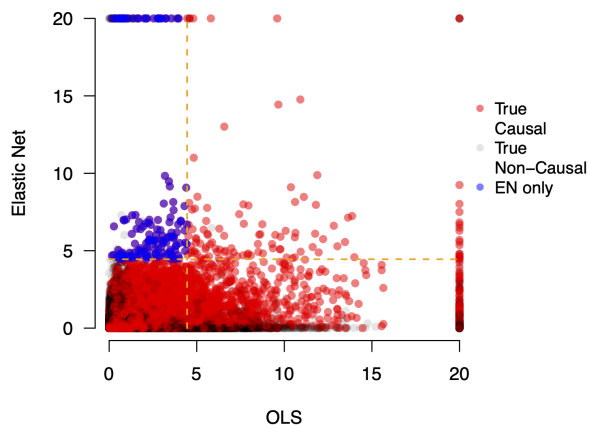

(D)  $N = 10,000$  with 10% Enriched Genes

Supplement: S19 Fig — Here, the narrow-sense heritability of the simulated quantitative traits is h2 = 0.2 and sample sizes are set to N = 5,000 in (A, B) and N = 10,000 in (C, D). In this simulation, traits were generated while using the top five principal components (PCs) of the genotype matrix as covariates. GWA summary statistics were computed by fitting a single-SNP univariate linear model (via ordinary least squares) without any control for the additional structure. Results are shown comparing the -log10 transformed gene-level P-values derived by gene-ε with Elastic Net (EN) regularization on the y-axis and without regularization (labeled as OLS) on the x-axis. The horizontal and vertical dashed lines are marked at the Bonferonni-corrected threshold P = 3.55×10^−5^ corrected for the 1,408 genes on chromosome 1 from the UK Biobank genotype data. True positive causal genes used to generate the synthetic phenotypes are colored in red, while non-causal genes are given in grey. Genes in the top right quadrant are selected by both approaches. Genes in the top left and bottom right quadrants are uniquely identified by gene-ε-EN and gene-ε-OLS, respectively. To illustrate the importance of regularization on SNP-level summary statistics, we highlight the true positive genes only identified by gene-ε-EN in blue. Each plot combines results from 100 simulated replicates (see S1 Text). (PDF) [file pgen.1008855.s019.pdf]

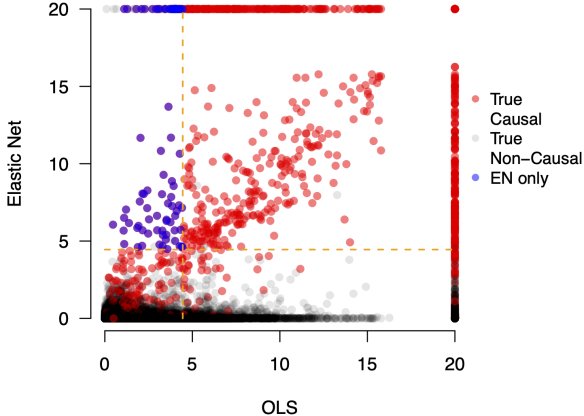

(A)  $N = 5,000$  with 1% Enriched Genes

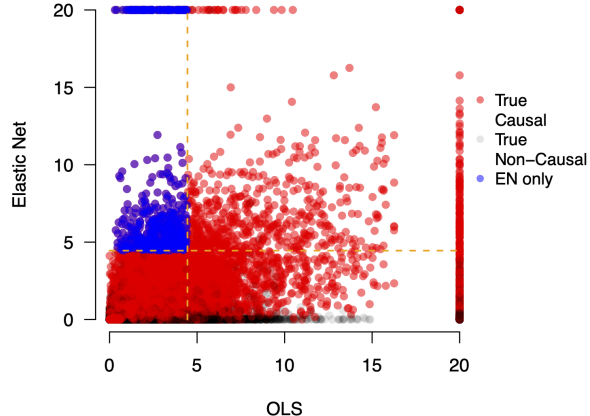

(B)  $N = 5,000$  with 10% Enriched Genes

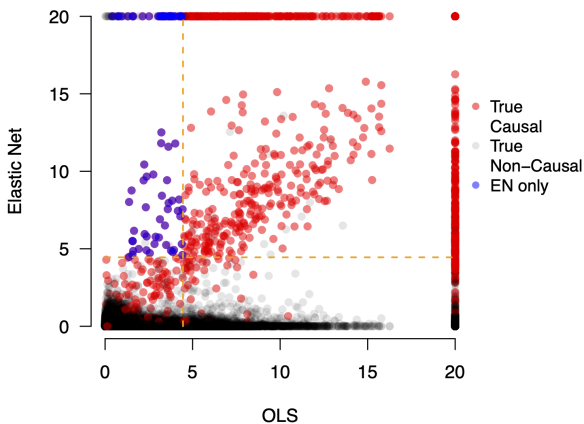

(C)  $N = 10,000$  with 1% Enriched Genes

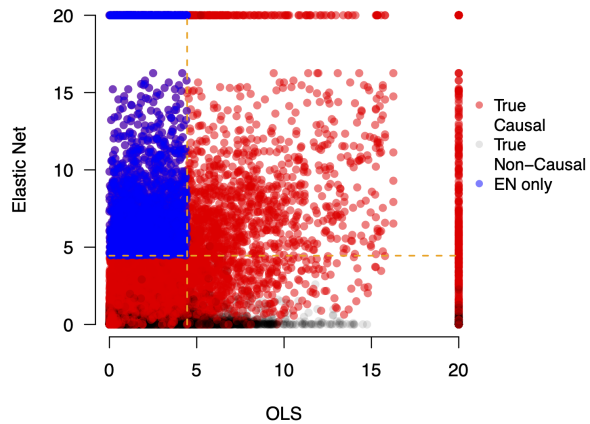

(D)  $N = 10,000$  with 10% Enriched Genes

Supplement: S20 Fig — Here, the narrow-sense heritability of the simulated quantitative traits is h2 = 0.6 and sample sizes are set to N = 5,000 in (A, B) and N = 10,000 in (C, D). In this simulation, traits were generated while using the top five principal components (PCs) of the genotype matrix as covariates. GWA summary statistics were computed by fitting a single-SNP univariate linear model (via ordinary least squares) without any control for the additional structure. Results are shown comparing the -log10 transformed gene-level P-values derived by gene-ε with Elastic Net (EN) regularization on the y-axis and without regularization (labeled as OLS) on the x-axis. The horizontal and vertical dashed lines are marked at the Bonferonni-corrected threshold P = 3.55×10^−5^ corrected for the 1,408 genes on chromosome 1 from the UK Biobank genotype data. True positive causal genes used to generate the synthetic phenotypes are colored in red, while non-causal genes are given in grey. Genes in the top right quadrant are selected by both approaches. Genes in the top left and bottom right quadrants are uniquely identified by gene-ε-EN and gene-ε-OLS, respectively. To illustrate the importance of regularization on SNP-level summary statistics, we highlight the true positive genes only identified by gene-ε-EN in blue. Each plot combines results from 100 simulated replicates (see S1 Text). (PDF) [file pgen.1008855.s020.pdf]

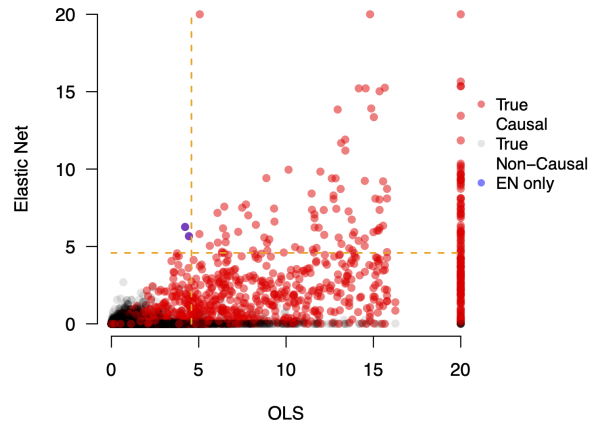

(A)  $N = 5,000$  with 1% Enriched Genes

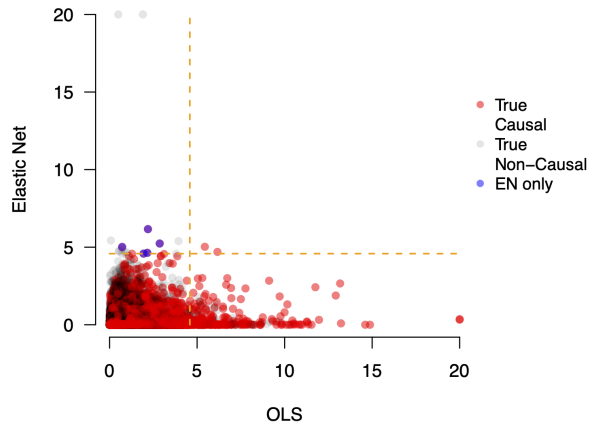

(B)  $N = 5,000$  with 10% Enriched Genes

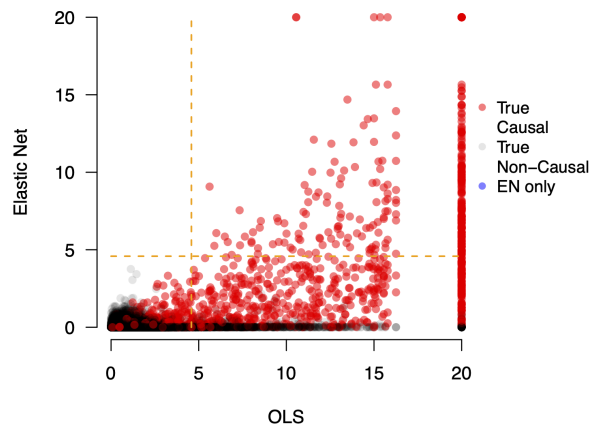

(C)  $N = 10,000$  with 1% Enriched Genes

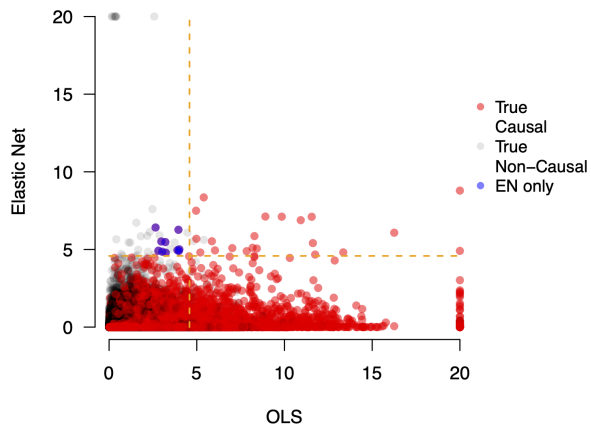

(D)  $N = 10,000$  with 10% Enriched Genes

Supplement: S21 Fig — Here, the narrow-sense heritability of the simulated quantitative traits is h2 = 0.2 and sample sizes are set to N = 5,000 in (A, B) and N = 10,000 in (C, D). In each case, standard GWA summary statistics were computed by fitting a single-SNP univariate linear model (via ordinary least squares). Results are shown comparing the -log10 transformed gene-level P-values derived by gene-ε with Elastic Net (EN) regularization on the y-axis and without regularization (labeled as OLS) on the x-axis. The horizontal and vertical dashed lines are marked at the Bonferonni-corrected threshold P = 2.61×10^−5^ corrected for the 1,916 genes on chromosome 1 from the UK Biobank genotype data. True positive causal genes used to generate the synthetic phenotypes are colored in red, while non-causal genes are given in grey. Genes in the top right quadrant are selected by both approaches. Genes in the top left and bottom right quadrants are uniquely identified by gene-ε-EN and gene-ε-OLS, respectively. To illustrate the importance of regularization on SNP-level summary statistics, we highlight the true positive genes only identified by gene-ε-EN in blue. Each plot combines results from 100 simulated replicates (see S1 Text). (PDF) [file pgen.1008855.s021.pdf]

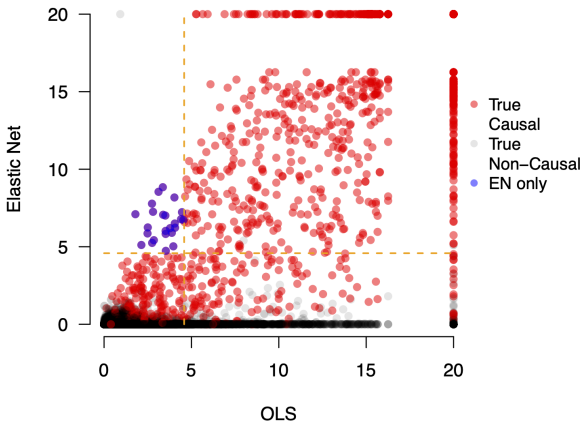

(A)  $N = 5,000$  with 1% Enriched Genes

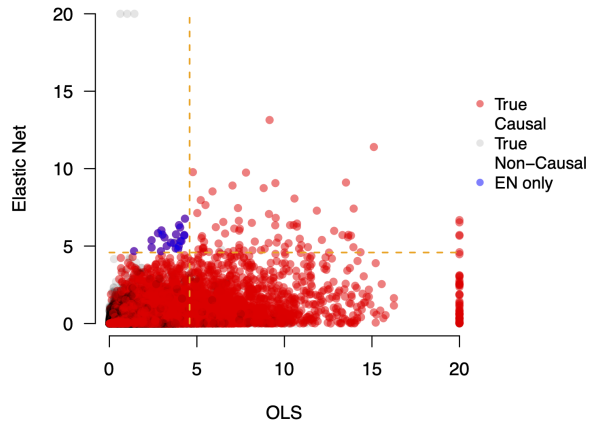

(B)  $N = 5,000$  with 10% Enriched Genes

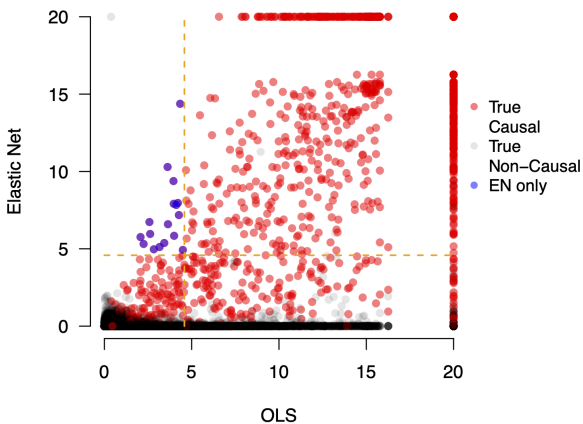

(C)  $N = 10,000$  with 1% Enriched Genes

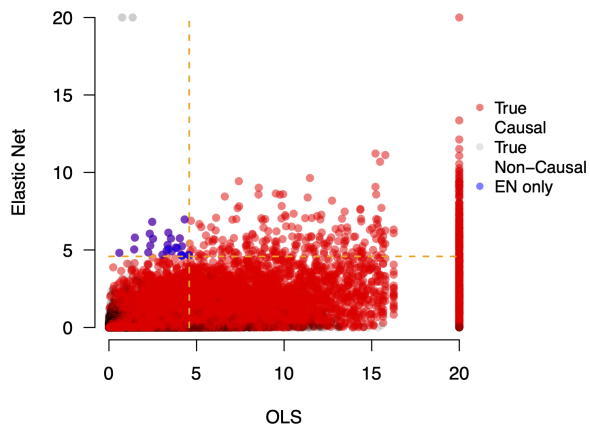

(D)  $N = 10,000$  with 10% Enriched Genes

Supplement: S22 Fig — Here, the narrow-sense heritability of the simulated quantitative traits is h2 = 0.6 and sample sizes are set to N = 5,000 in (A, B) and N = 10,000 in (C, D). In each case, standard GWA summary statistics were computed by fitting a single-SNP univariate linear model (via ordinary least squares). Results are shown comparing the -log10 transformed gene-level P-values derived by gene-ε with Elastic Net (EN) regularization on the y-axis and without regularization (labeled as OLS) on the x-axis. The horizontal and vertical dashed lines are marked at the Bonferonni-corrected threshold P = 2.61×10^−5^ corrected for the 1,916 genes on chromosome 1 from the UK Biobank genotype data. True positive causal genes used to generate the synthetic phenotypes are colored in red, while non-causal genes are given in grey. Genes in the top right quadrant are selected by both approaches. Genes in the top left and bottom right quadrants are uniquely identified by gene-ε-EN and gene-ε-OLS, respectively. To illustrate the importance of regularization on SNP-level summary statistics, we highlight the true positive genes only identified by gene-ε-EN in blue. Each plot combines results from 100 simulated replicates (see S1 Text). (PDF) [file pgen.1008855.s022.pdf]

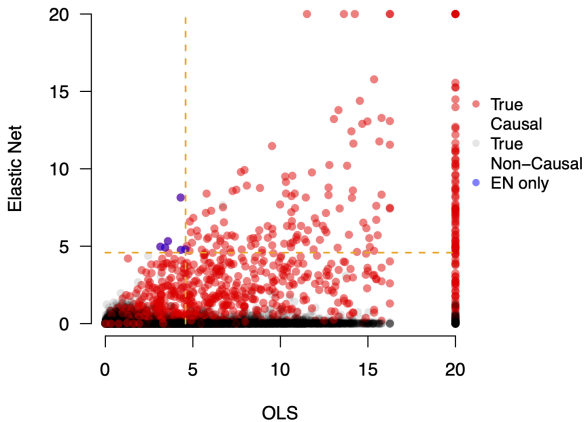

(A)  $N = 5,000$  with 1% Enriched Genes

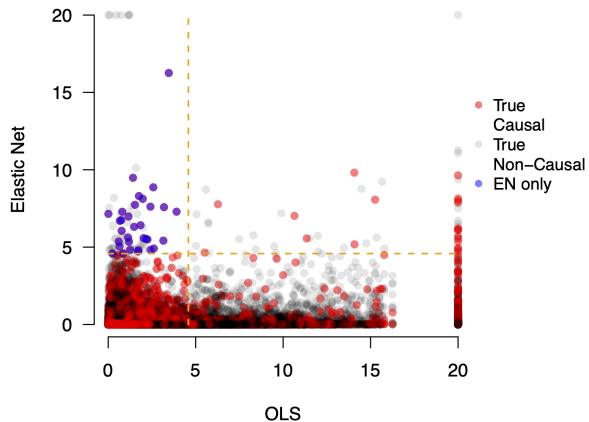

(B)  $N = 5,000$  with 10% Enriched Genes

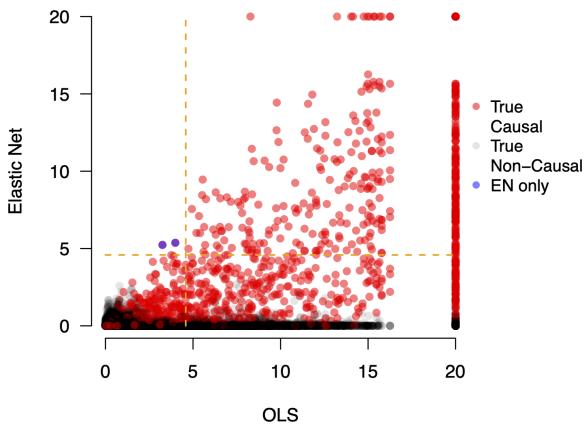

(C)  $N = 10,000$  with 1% Enriched Genes

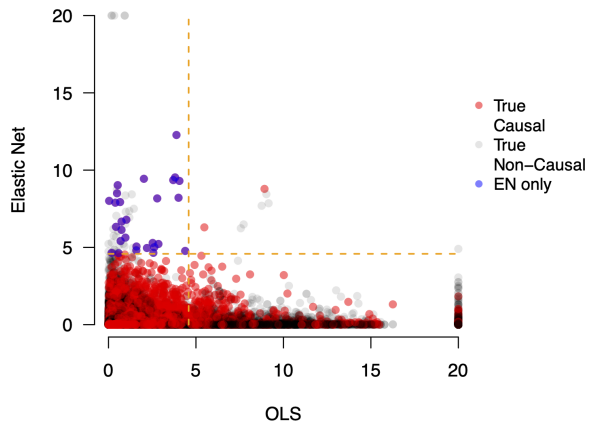

(D)  $N = 10,000$  with 10% Enriched Genes

Supplement: S23 Fig — Here, the narrow-sense heritability of the simulated quantitative traits is h2 = 0.2 and sample sizes are set to N = 5,000 in (A, B) and N = 10,000 in (C, D). In this simulation, traits were generated while using the top five principal components (PCs) of the genotype matrix as covariates. GWA summary statistics were computed by fitting a single-SNP univariate linear model (via ordinary least squares) without any control for the additional structure. Results are shown comparing the -log10 transformed gene-level P-values derived by gene-ε with Elastic Net (EN) regularization on the y-axis and without regularization (labeled as OLS) on the x-axis. The horizontal and vertical dashed lines are marked at the Bonferonni-corrected threshold P = 2.61×10^−5^ corrected for the 1,916 genes on chromosome 1 from the UK Biobank genotype data. True positive causal genes used to generate the synthetic phenotypes are colored in red, while non-causal genes are given in grey. Genes in the top right quadrant are selected by both approaches. Genes in the top left and bottom right quadrants are uniquely identified by gene-ε-EN and gene-ε-OLS, respectively. To illustrate the importance of regularization on SNP-level summary statistics, we highlight the true positive genes only identified by gene-ε-EN in blue. Each plot combines results from 100 simulated replicates (see S1 Text). (PDF) [file pgen.1008855.s023.pdf]

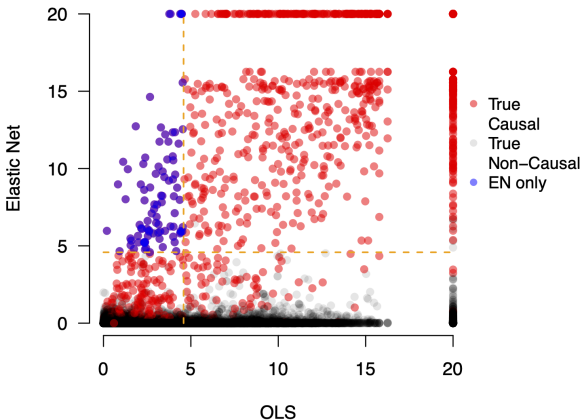

(A)  $N = 5,000$  with 1% Enriched Genes

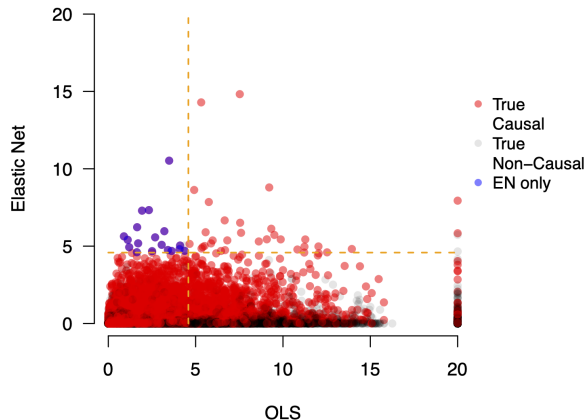

(B)  $N = 5,000$  with 10% Enriched Genes

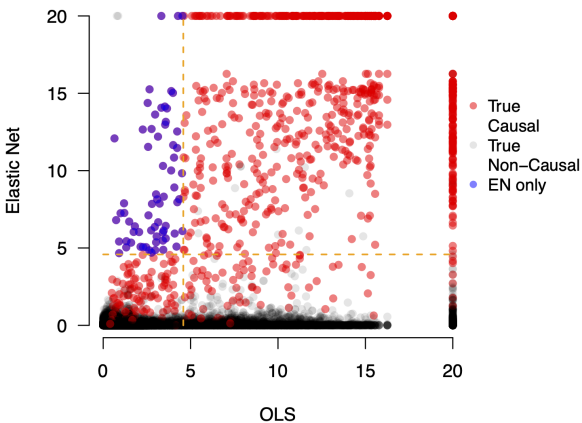

(C)  $N = 10,000$  with 1% Enriched Genes

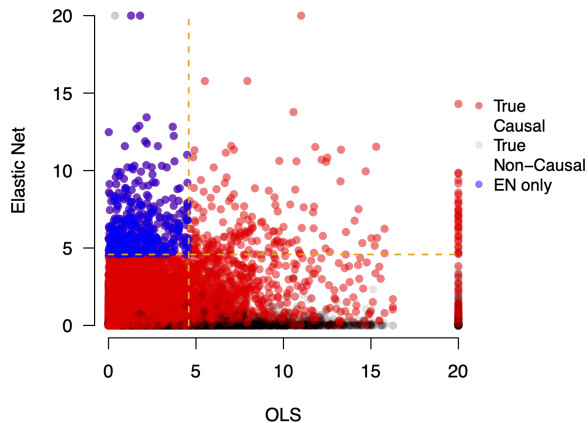

(D)  $N = 10,000$  with 10% Enriched Genes

Supplement: S24 Fig — Here, the narrow-sense heritability of the simulated quantitative traits is h2 = 0.6 and sample sizes are set to N = 5,000 in (A, B) and N = 10,000 in (C, D). In this simulation, traits were generated while using the top five principal components (PCs) of the genotype matrix as covariates. GWA summary statistics were computed by fitting a single-SNP univariate linear model (via ordinary least squares) without any control for the additional structure. Results are shown comparing the -log10 transformed gene-level P-values derived by gene-ε with Elastic Net (EN) regularization on the y-axis and without regularization (labeled as OLS) on the x-axis. The horizontal and vertical dashed lines are marked at the Bonferonni-corrected threshold P = 2.61×10^−5^ corrected for the 1,916 genes on chromosome 1 from the UK Biobank genotype data. True positive causal genes used to generate the synthetic phenotypes are colored in red, while non-causal genes are given in grey. Genes in the top right quadrant are selected by both approaches. Genes in the top left and bottom right quadrants are uniquely identified by gene-ε-EN and gene-ε-OLS, respectively. To illustrate the importance of regularization on SNP-level summary statistics, we highlight the true positive genes only identified by gene-ε-EN in blue. Each plot combines results from 100 simulated replicates (see S1 Text). (PDF) [file pgen.1008855.s024.pdf]
