# Supplementary material for: Estimation of non-null SNP effect size distributions enables the detection of enriched genes underlying complex traits
Source: PLoS Genet. 2020 Jun 15;16(6):e1008855. doi: 10.1371/journal.pgen.1008855 (PMC7316356; doi:10.1371/journal.pgen.1008855)
Supplement: S25 Fig — Body height has been estimated to have a narrow-sense heritability h2 in the range of 0.45 to 0.80 [6, 31–39]; while, MPV has been estimated to have h2 between 0.50 and 0.70 [33, 34, 58]. Manhattan plots of gene-ε gene-level association P-values using Elastic Net regularized effect sizes for (A) body height and (B) MPV. The purple dashed line indicates a log-transformed Bonferroni-corrected significance threshold (P = 2.83×10^−6^ correcting for 17,680 autosomal genes analyzed). We color code all significant genes identified by gene-ε in orange, and annotate genes overlapping with the database of Genotypes and Phenotypes (dbGaP). In (C) and (D), we conduct gene set enrichment analysis using Enrichr [46, 59] to identify dbGaP categories enriched for significant gene-level associations reported by gene-ε. We highlight categories with Q-values (i.e., false discovery rates) less than 0.05 and annotate corresponding genes in the Manhattan plots in (A) and (B), respectively. For height, the most enriched dbGAP category is “Body Height”, with 5 of the genes identified by gene-ε appearing in this category. For MPV, the four significant dbGAP categories are “Platelet Count”, “Behcet Syndrome”, “Psoriasis”, and “Face”—all of which have been connected to trait [57, 60, 61, 112, 113]. (PDF) [file pgen.1008855.s025.pdf]

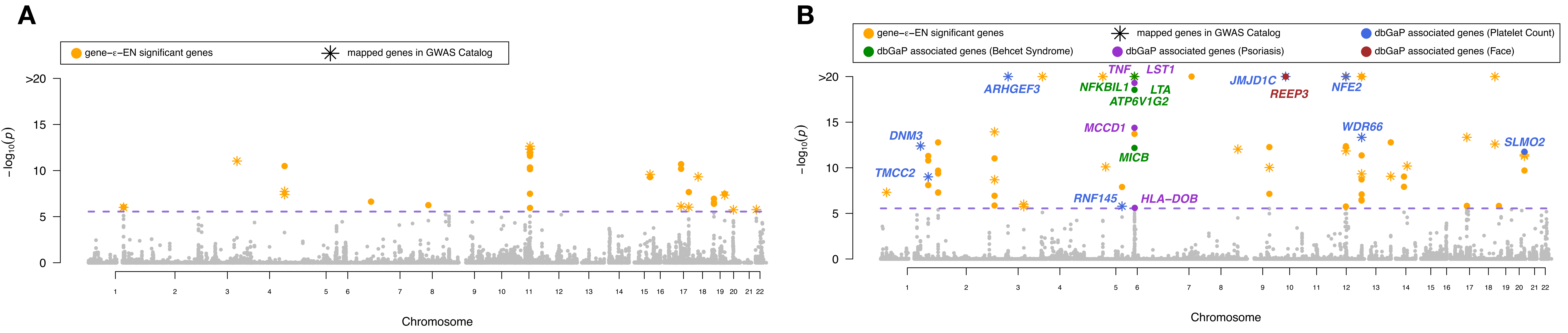

C

	p value	q value	Odds.ratio	Combined score	# of sig. genes in dbGaP
Body Height	5.20e-04	1.79e-01	7.42	56.12	5
Macular Degeneration	1.14e-02	1.97e-01	14.53	98.43	3
Prion Diseases	1.22e-02	1.00	81.63	359.78	1
Neuroblastoma	1.35e-02	1.00	11.32	48.73	2
Smoking	2.08e-02	1.00	47.62	184.41	1
Stroke	9.07e-02	1.00	3.95	9.49	2
Prostatic Neoplasms	1.23e-01	1.00	7.62	15.95	1
Creatinine	1.49e-01	1.00	6.21	11.82	1
Respiratory Function Tests	2.33e-01	1.00	3.78	5.51	1
—	—	—	—	—	—

D

	p value	q value	Odds.ratio	Combined score	# of sig. genes in dbGaP
Platelet Count	2.16e-11	7.45e-09	40.04	983.32	8
Behcet Syndrome	2.59e-06	4.47e-04	22.90	294.64	5
Psoriasis	1.74e-05	2.00e-03	25.74	282.13	4
Face	8.06e-05	6.96e-03	135.14	1273.71	2
Diabetes Mellitus, Type 1	2.71e-03	1.87e-01	10.81	63.89	3
Dengue Hemorrhagic Fever	1.11e-02	6.36e-01	90.09	405.81	1
Hearing Loss	1.11e-02	5.45e-01	90.09	405.81	1
Erythrocytes	1.59e-02	6.85e-01	10.40	43.05	2
Body Mass Index	2.29e-02	8.78e-01	3.09	11.68	5
Arteries	4.30e-02	1.00	6.07	19.11	2
