# Supplementary material for: Estimation of non-null SNP effect size distributions enables the detection of enriched genes underlying complex traits
Source: PLoS Genet. 2020 Jun 15;16(6):e1008855. doi: 10.1371/journal.pgen.1008855 (PMC7316356; doi:10.1371/journal.pgen.1008855)
Supplement: S27 Fig — MCV has been estimated to have a narrow-sense heritability h2 in the range of 0.20 to 0.60 [33, 34, 117, 118]. Manhattan plots of gene-ε gene-level association P-values using Elastic Net regularized effect sizes when gene boundaries are defined by (A) using UCSC annotations directly, and (B) augmenting the gene boundaries by adding SNPs within a ±50kb buffer. The purple dashed line indicates a log-transformed Bonferroni-corrected significance threshold (P = 3.49×10^−6^ and P = 2.83×10^−6^ correcting for the 14,322 and 17,680 autosomal genes analyzed, respectively). We color code all significant genes identified by gene-ε in orange, and annotate genes previously associated with MCV in the database of Genotypes and Phenotypes (dbGaP). In (C) and (D), we conduct gene set enrichment analysis using Enrichr [46, 59] to identify dbGaP categories enriched for significant gene-level associations reported by gene-ε. We highlight categories with Q-values (i.e., false discovery rates) less than 0.05 and annotate corresponding genes in the Manhattan plots in (A) and (B), respectively. The dbGAP categories significantly enriched for gene-level associations with MCV included “Transferrin”, “Erythrocyte Indices”, “Hematocrit”, “Narcolepsy”, and “Iron”—all of which have been connected to trait [50–57]. (PDF) [file pgen.1008855.s027.pdf]

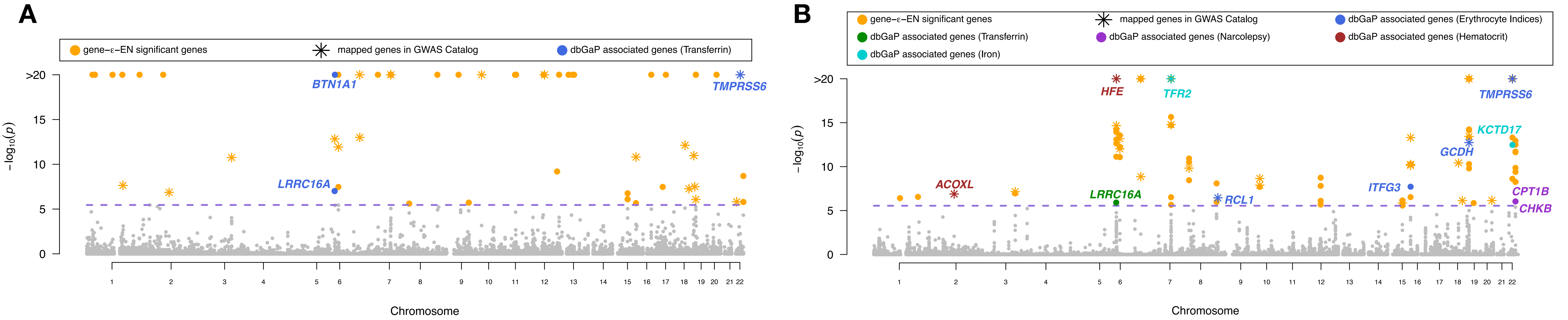

C

	p value	q value	Odds.ratio	Combined score	# of sig. genes in dbGaP
Transferrin	3.56e-06	1.23e-03	95.69	1200.47	3
Hematocrit	7.02e-04	1.21e-01	17.26	125.30	3
Iron-Regulatory Proteins	8.53e-03	9.80e-01	16.96	557.27	1
Erythrocyte Indices	9.30e-03	8.02e-01	13.76	64.36	2
Iron	9.67e-03	6.67e-01	6.84	31.71	3
Platelet Count	1.04e-02	5.97e-01	13.00	59.35	2
Esophageal Neoplasms	2.54e-02	1.00	38.99	143.25	1
Leukemia, Lymphocytic, Chronic, B-Cell	2.81e-02	1.00	35.09	125.28	1
Glomerulonephritis, IGA	3.09e-02	1.00	31.90	110.89	1
Hemoglobin A, Glycosylated	5.16e-02	1.00	5.48	16.25	2

D

	p value	q value	Odds.ratio	Combined score	# of sig. genes in dbGaP
Erythrocyte Indices	3.48e-06	1.20e-03	21.55	270.84	5
Transferrin	1.46e-05	2.53e-03	59.94	667.23	3
Narcolepsy	6.12e-05	7.04e-03	146.52	1421.34	2
Hematocrit	1.72e-04	1.48e-02	14.41	124.95	4
Iron	6.93e-04	4.78e-02	7.14	51.91	5
Ferritins	1.36e-02	7.81e-01	73.26	314.91	1
carbohydrate-deficient transferrin	1.36e-02	6.70e-01	73.26	314.91	1
Iron-Regulatory Proteins	1.36e-02	5.86e-01	73.26	314.91	1
Blood Pressure	1.75e-02	6.70e-01	2.90	11.75	6
Prostate-Specific Antigen	1.81e-02	6.24e-01	54.95	220.50	1
