# Supplementary material for: Estimation of non-null SNP effect size distributions enables the detection of enriched genes underlying complex traits
Source: PLoS Genet. 2020 Jun 15;16(6):e1008855. doi: 10.1371/journal.pgen.1008855 (PMC7316356; doi:10.1371/journal.pgen.1008855)
Supplement: S28 Fig — PLC has been estimated to have a narrow-sense heritability h2 ranging from 0.55 to 0.80 [33, 34, 58]. Manhattan plots of gene-ε gene-level association P-values using Elastic Net regularized effect sizes when gene boundaries are defined by (A) using UCSC annotations directly, and (B) augmenting the gene boundaries by adding SNPs within a ±50kb buffer. The purple dashed line indicates a log-transformed Bonferroni-corrected significance threshold (P = 3.49×10^−6^ and P = 2.83×10^−6^ correcting for the 14,322 and 17,680 autosomal genes analyzed, respectively). We color code all significant genes identified by gene-ε in orange, and annotate genes previously associated with PLC in the database of Genotypes and Phenotypes (dbGaP). In (C) and (D), we conduct gene set enrichment analysis using Enrichr [46, 59] to identify dbGaP categories enriched for significant gene-level associations reported by gene-ε. We highlight categories with Q-values (i.e., false discovery rates) less than 0.05 and annotate corresponding genes in the Manhattan plots in (A) and (B), respectively. The most significant dbGAP category is “Platelet Count” for both SNP-to-gene annotation schemes. The other significant dbGAP category was “Smoking” which has been previously connected to PLC [61, 119, 120]. (PDF) [file pgen.1008855.s028.pdf]

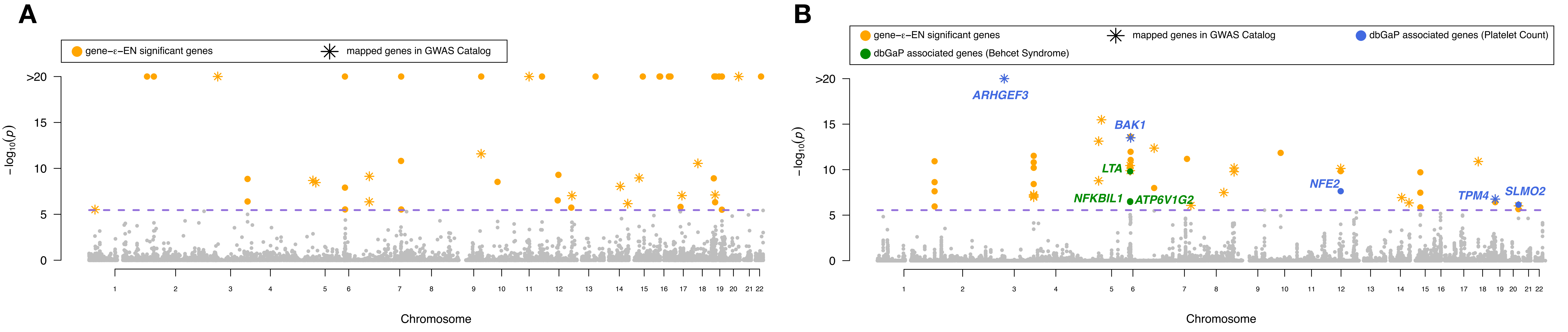

C

	p value	q value	Odds.ratio	Combined score	# of sig. genes in dbGaP
Platelet Count	2.95e-04	1.02e-01	23.15	188.14	3
Smoking	3.67e-04	6.32e-02	69.44	549.39	2
Hearing Loss	7.18e-03	8.26e-01	138.89	685.56	1
Esophageal Neoplasms	2.14e-02	1.00	46.30	177.98	1
Macular Degeneration	3.26e-02	1.00	7.06	24.17	2
Cardiomegaly	3.54e-02	1.00	27.78	92.80	1
Pulse	8.07e-02	1.00	11.90	29.96	1
Body Mass Index	8.72e-02	1.00	2.86	6.98	3
Arthritis, Rheumatoid	1.32e-01	1.00	7.06	14.28	1
Coronary Disease	1.79e-01	1.00	5.08	8.74	1

D

	p value	q value	Odds.ratio	Combined score	# of sig. genes in dbGaP
Platelet Count	2.07e-07	7.13e-05	37.79	581.69	5
Smoking	3.82e-04	6.59e-02	68.03	535.37	2
Behcet Syndrome	4.08e-04	4.69e-02	20.75	161.97	3
Psoriasis	4.76e-03	4.10e-01	19.44	103.95	2
Erythrocyte Indices	6.94e-03	4.79e-01	16.01	79.55	2
Hearing Loss	7.33e-03	4.22e-01	136.05	668.77	1
Testicular Neoplasms	1.22e-02	6.01e-01	81.63	359.76	1
Cardiomegaly	3.61e-02	1.00	27.21	90.35	1
Heart Rate	5.54e-02	1.00	5.27	15.24	2
Aorta	8.01e-02	1.00	12.00	30.31	1
