# Supplementary material for: Estimation of non-null SNP effect size distributions enables the detection of enriched genes underlying complex traits
Source: PLoS Genet. 2020 Jun 15;16(6):e1008855. doi: 10.1371/journal.pgen.1008855 (PMC7316356; doi:10.1371/journal.pgen.1008855)
Supplement: S29 Fig — WHR has been estimated to have a narrow-sense heritability h2 ranging from 0.10 to 0.25 [31, 33, 35, 42, 45, 121]. Manhattan plots of gene-ε gene-level association P-values using Elastic Net regularized effect sizes when gene boundaries are defined by (A) using UCSC annotations directly, and (B) augmenting the gene boundaries by adding SNPs within a ±50kb buffer. The purple dashed line indicates a log-transformed Bonferroni-corrected significance threshold (P = 3.49×10^−6^ and P = 2.83×10^−6^ correcting for the 14,322 and 17,680 autosomal genes analyzed, respectively). We color code all significant genes identified by gene-ε in orange, and annotate genes previously associated with WHR in the database of Genotypes and Phenotypes (dbGaP). In (C) and (D), we conduct gene set enrichment analysis using Enrichr [46, 59] to identify dbGaP categories enriched for significant gene-level associations reported by gene-ε in (A) and (B), respectively. While many of the scored categories are biologically related to WHR (e.g., “Body Mass Index”, “Adiposity”, and “Inflammatory Bowel Diseases”) [122, 123], none of them had Q-values (i.e., false discovery rates) less than 0.05. (PDF) [file pgen.1008855.s029.pdf]

A

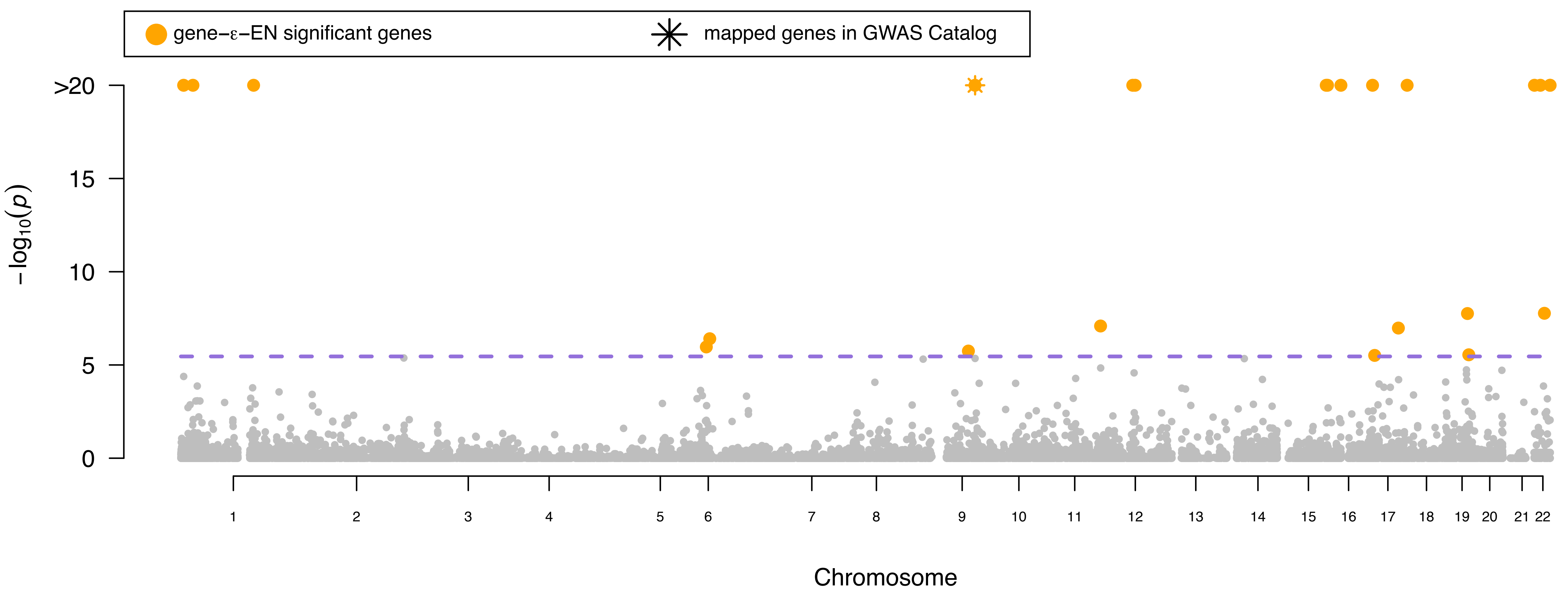

B

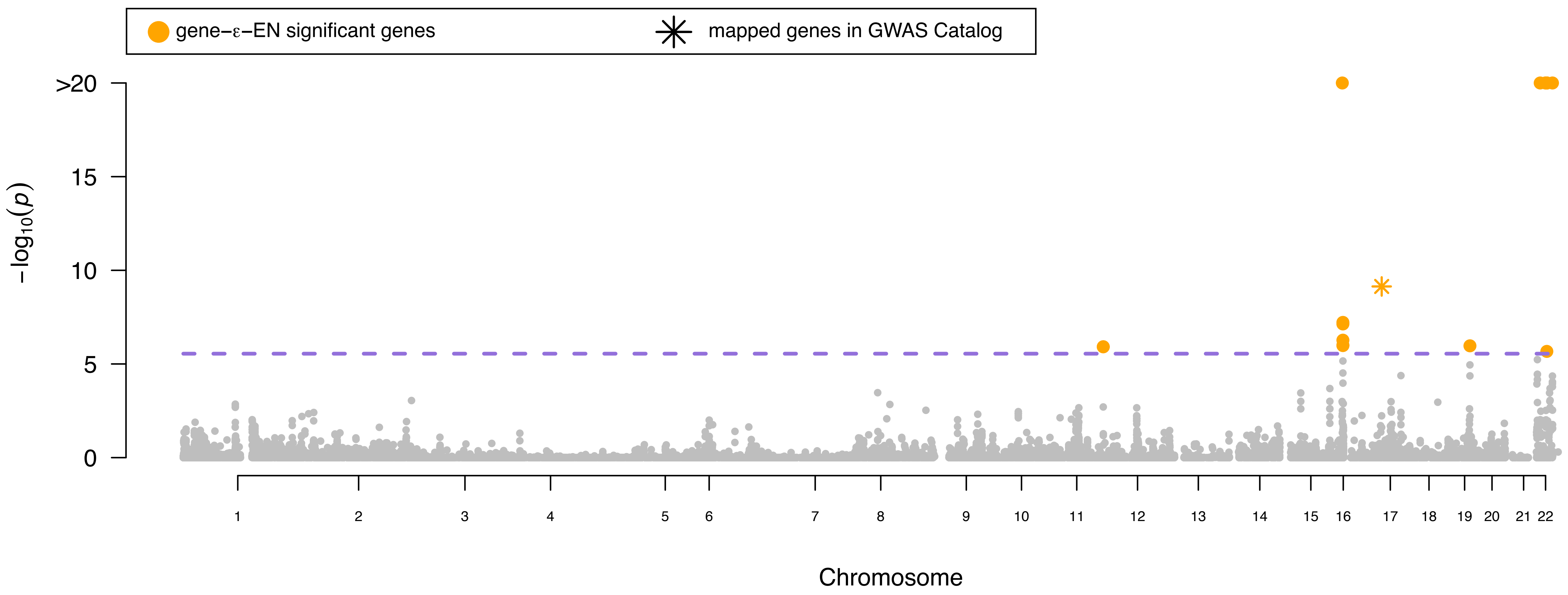

C

	p value	*q* value	Odds.ratio	Combined score	# of sig. genes in dbGaP
Adiposity	4.99e-03	1.00	200.00	1060.03	1
Glomerulonephritis, IGA	1.37e-02	1.00	72.73	312.20	1
Metabolic Syndrome X	2.84e-02	1.00	34.78	123.91	1
Inflammatory Bowel Diseases	4.29e-02	1.00	22.86	71.99	1
Schizophrenia	9.77e-02	1.00	9.76	22.69	1
Body Mass Index	1.03e-01	1.00	3.66	8.33	2
Macular Degeneration	1.38e-01	1.00	6.78	13.45	1
Stroke	3.05e-01	1.00	2.77	3.29	1
—	—	—	—	—	—
—	—	—	—	—	—

D

	p value	*q* value	Odds.ratio	Combined score	# of sig. genes in dbGaP
Dermatitis, Atopic	3.79e-03	1.00	263.16	1466.8	1
gamma-Glutamyltransferase	2.07e-02	1.00	47.85	185.52	1
Alkaline Phosphatase	2.16e-02	1.00	45.77	175.44	1
Inflammatory Bowel Diseases	3.27e-02	1.00	30.08	102.83	1
Body Mass Index	3.43e-01	1.00	2.41	2.58	1
—	—	—	—	—	—
—	—	—	—	—	—
—	—	—	—	—	—
—	—	—	—	—	—
—	—	—	—	—	—
