# Supplementary material for: Estimation of non-null SNP effect size distributions enables the detection of enriched genes underlying complex traits
Source: PLoS Genet. 2020 Jun 15;16(6):e1008855. doi: 10.1371/journal.pgen.1008855 (PMC7316356; doi:10.1371/journal.pgen.1008855)
Supplement: S13 Table — In this simulation, traits were generated while using the top five principal components (PCs) of the genotype matrix as covariates. GWA summary statistics were computed by fitting a single-SNP univariate linear model (via ordinary least squares) without any control for the additional structure. We show the power of gene-ε to identify enriched genes under the Bonferonni-corrected threshold P = 2.61×10^−5^, corrected for 1,916 genes simulated using chromosome 1 from the UK Biobank genotype data (see S1 Text). Results for gene-ε are shown with LASSO, Elastic Net (EN), and Ridge Regression (RR) regularizations. We also show the power of gene-ε without regularization to illustrate the importance of this step (OLS). Additionally, we compare the performance gene-ε with five existing methods: PEGASUS [12], VEGAS [7], RSS [14], SKAT [20], and MAGMA [10]. The last is a Bayesian method and is evaluated based on the “median probability criterion” (i.e., posterior enrichment probability of a gene is greater than 0.5). All results are based on 100 replicates and standard deviations of the estimates across runs are given in the parentheses. Approaches with the greatest power are bolded in purple, while methods with the lowest FDR is bolded in blue. (PDF) [file pgen.1008855.s042.pdf]

		gene-ε Approaches			
**Causal Genes**	**Metric**	**OLS**	**RR**	**EN**	**LASSO**
1%	Power	0.431 (0.117)	0.427 (0.137)	0.155 (0.073)	0.158 (0.073)
	FDR	0.838 (0.128)	0.577 (0.286)	**0.004 (0.035)**	0.014 (0.081)
10%	Power	0.026 (0.089)	**0.079 (0.030)**	0.027 (0.035)	0.009 (0.021)
	FDR	0.903 (0.091)	**0.571 (0.125)**	0.624 (0.392)	0.705 (0.430)

		Other Methods				
**Causal Genes**	**Metric**	**PEGASUS**	**VEGAS**	**RSS**	**SKAT**	**MAGMA**
1%	Power	0.463 (0.116)	**0.556 (0.117)**	0.520 (0.110)	0.387 (0.110)	0.500 (0.110)
	FDR	0.847 (0.115)	0.828 (0.114)	0.608 (0.133)	0.858 (0.115)	0.846 (0.105)
10%	Power	0.027 (0.013)	0.016 (0.011)	0.006 (0.006)	0.022 (0.012)	0.020 (0.011)
	FDR	0.868 (0.094)	0.921 (0.081)	0.898 (0.103)	0.886 (0.091)	0.896 (0.091)
