# Supplementary material for: Estimation of non-null SNP effect size distributions enables the detection of enriched genes underlying complex traits
Source: PLoS Genet. 2020 Jun 15;16(6):e1008855. doi: 10.1371/journal.pgen.1008855 (PMC7316356; doi:10.1371/journal.pgen.1008855)
Supplement: S17 Table — Here, quantitative traits are simulated with just noise randomly drawn from standard normal distributions. This represents the scenario in which all SNPs are non-causal and satisfy the conventional null hypothesis H0: βj = 0. GWA summary statistics were computed by fitting a single-SNP univariate linear model (via ordinary least squares). Each table entry lists the mean type I error rate estimates for the four gene-ε modeling approaches—which is computed as the proportion of P-values under some significance level α. Empirical size for the analyses used significance levels of α = 0.05, 0.01, 0.001, and 2.61×10^−5^ (the Bonferonni-corrected threshold), respectively. Sample sizes of the individual-level data (used to derive the summary statistics), were set to N = 5,000 and 10,000 observations. These results are based on 100 simulated datasets and the standard errors across the replicated are included in the parentheses. Overall, gene-ε controls the type I error rate for reasonably sized datasets, and can be slightly conservative when the sample size is small and the GWA summary statistics are less precise/more inflated. (PDF) [file pgen.1008855.s046.pdf]

		Significance Level			
**Sample Size**	**gene-ε Approach**	$\alpha = 0.05$	$\alpha = 0.01$	$\alpha = 0.001$	$\alpha = 2.61 \times 10^{-5}$
$N = 5{,}000$	OLS	0.0481 (0.0103)	0.0091 (0.0038)	0.0008 (0.0010)	0.0000 (0.0001)
	Ridge Regression	0.0082 (0.0024)	0.0065 (0.0020)	0.0056 (0.0018)	0.0000 (0.0003)
	Elastic Net	0.0035 (0.0094)	0.0013 (0.0045)	0.0004 (0.0016)	0.0000 (0.0001)
	LASSO	0.0043 (0.0093)	0.0015 (0.0043)	0.0004 (0.0013)	0.0000 (0.0001)
$N = 10{,}000$	OLS	0.0486 (0.0109)	0.0095 (0.0034)	0.0008 (0.0008)	0.0000 (0.0000)
	Ridge Regression	0.0067 (0.0029)	0.0050 (0.0031)	0.0044 (0.0033)	0.0000 (0.0003)
	Elastic Net	0.0009 (0.0028)	0.0004 (0.0009)	0.0000 (0.0002)	0.0000 (0.0001)
	LASSO	0.0007 (0.0026)	0.0002 (0.0009)	0.0000 (0.0002)	0.0000 (0.0001)
