# Supplementary material for: Estimation of non-null SNP effect size distributions enables the detection of enriched genes underlying complex traits
Source: PLoS Genet. 2020 Jun 15;16(6):e1008855. doi: 10.1371/journal.pgen.1008855 (PMC7316356; doi:10.1371/journal.pgen.1008855)
Supplement: S25 Table — Here, we report the way different regularizations in gene-ε characterize ε-genic effects in complex traits. Results are shown for Elastic Net (which is highlighted in the main text), as well as for LASSO and Ridge Regression. We also show results when no shrinkage is applied to illustrate the importance of this step (denoted by OLS). In the three former cases, we regress the GWA SNP-level effect size estimates onto chromosome-specific LD matrices to derive a regularized set of summary statistics β˜. gene-ε assumes a reformulated null distribution of SNP-level effects β˜j∼N(0,σε2), where σε2 is the SNP-level null threshold and represents the maximum proportion of phenotypic variance explained (PVE) by a spurious or non-associated SNP. We used an EM-algorithm with 100 iterations to fit K-mixture Gaussian models over the regularized effect sizes to estimate σε2. Here, each mixture component had distinctively smaller variances (σ12>⋯>σK2; with the K-th component fixed at σK2=0), and the number of total mixture components K was chosen based on a grid of values where the best model yielded the highest Bayesian Information Criterion (BIC). We assume associated SNPs appear in the first component, non-associated SNPs appear in the last component, and null SNPs with spurious effects fell in between (i.e., σε2=σ22). Thus, a SNP is considered to have some level of association with a trait if E[βj2]>σK2=0; while a SNP is considered “causal” if E[βj2]>σ22. Column 3 gives the K used for each trait. Column 4 and 5 detail the percentage of associated and causal SNPs, respectively. The last column gives the mean threshold for ε-genic effects across the chromosomes. (PDF) [file pgen.1008855.s054.pdf]

gene-ε Approach	Trait	# Mix. Comp.	% Associated SNPs	% Causal SNPs	ε-genic Threshold (σ_2^2)
Elastic Net	Height	7	11.65%	1.27%	1.90×10^{-5}
	BMI	6	16.61%	7.60%	2.73×10^{-5}
	MCV	8	11.60%	0.21%	3.48×10^{-5}
	MPV	8	5.47%	0.20%	3.37×10^{-5}
	PLC	7	10.33%	0.39%	3.68×10^{-5}
	WHR	8	14.38%	7.20%	3.67×10^{-5}
LASSO	Height	8	10.66%	0.67%	2.29×10^{-5}
	BMI	6	16.57%	7.35%	2.71×10^{-5}
	MCV	7	9.94%	0.23%	3.56×10^{-5}
	MPV	9	4.51%	0.20%	3.32×10^{-5}
	PLC	8	8.57%	0.26%	3.80×10^{-4}
	WHR	6	15.27%	7.05%	3.29×10^{-4}
Ridge Regression	Height	5	47.73%	13.93%	5.67×10^{-7}
	BMI	5	38.53%	17.98%	1.49×10^{-7}
	MCV	5	45.28%	9.79%	8.76×10^{-6}
	MPV	9	34.42%	2.69%	1.13×10^{-7}
	PLC	5	46.14%	8.59%	8.71×10^{-6}
	WHR	5	42.09%	19.12%	1.31×10^{-7}
OLS	Height	5	49.08%	6.36%	3.08×10^{-5}
	BMI	3	54.54%	25.25%	3.31×10^{-5}
	MCV	9	34.39%	1.96%	5.55×10^{-5}
	MPV	9	36.07%	2.16%	6.74×10^{-5}
	PLC	9	36.95%	1.84%	6.28×10^{-5}
	WHR	3	50.03%	23.81%	3.49×10^{-5}
