# Supplementary material for: Estimation of non-null SNP effect size distributions enables the detection of enriched genes underlying complex traits
Source: PLoS Genet. 2020 Jun 15;16(6):e1008855. doi: 10.1371/journal.pgen.1008855 (PMC7316356; doi:10.1371/journal.pgen.1008855)
Supplement: S26 Table — Traits include: height; body mass index (BMI); mean corpuscular volume (MCV); mean platelet volume (MPV); platelet count (PLC); and waist-hip ratio (WHR). Here, we list the number of significant genes found when using gene-ε with various regularization strategies, as well as the number of dbGAP categories enriched for significant genes identified by gene-ε. We also assess how well these results overlap with the gene-ε -EN findings that were reported in the main text. Significant genes were determined by using a Bonferroni-corrected P-value threshold (in our analyses, P = 0.05/13029 autosomal genes = 3.84×10^−6^). Enriched dbGAP categories were those with Enrichr Q-values (i.e., false discovery rates) less than 0.05. (PDF) [file pgen.1008855.s055.pdf]

	Trait	OLS	Ridge Regression	LASSO	Elastic Net
# Sig. Genes	Height	501	8	65	67
	BMI	640	8	42	40
	MCV	318	10	62	78
	MPV	326	29	62	66
	PLC	289	15	54	52
	WHR	677	6	49	22
% Sig. Gene Overlap w/ Elastic Net	Height	7.78%	37.50%	69.23%	—
	BMI	2.19%	12.50%	64.29%	—
	MCV	12.89%	60.00%	70.97%	—
	MPV	13.80%	51.70%	83.87%	—
	PLC	11.42%	46.67%	75.93%	—
	WHR	1.18%	0.00%	20.41%	—
# Enriched dbGaP Categories	Height	1	1	0	1
	BMI	33	16	0	0
	MCV	1	3	1	1
	MPV	6	1	2	2
	PLC	2	3	1	2
	WHR	23	3	0	0
% Enriched dbGaP Overlap w/ Elastic Net	Height	100.00% (Body Height)	100.00% (Body Height)	0.00%	—
	BMI	0.00%	0.00%	0.00%	—
	MCV	100.00% (Erythrocyte Indices)	33.33% (Erythrocyte Indices)	100.00% (Erythrocyte Indices)	—
	MPV	16.67% (Platelet Count)	100.00% (Platelet Count)	100.00% (Platelet Count; Face)	—
	PLC	50.00% (Platelet Count)	33.33% (Platelet Count)	100.00% (Platelet Count)	—
	WHR	0.00%	0.00%	0.00%	—
