# Supplementary material for: Estimation of non-null SNP effect size distributions enables the detection of enriched genes underlying complex traits
Source: PLoS Genet. 2020 Jun 15;16(6):e1008855. doi: 10.1371/journal.pgen.1008855 (PMC7316356; doi:10.1371/journal.pgen.1008855)
Supplement: S27 Table — Traits include: height; body mass index (BMI); mean corpuscular volume (MCV); mean platelet volume (MPV); platelet count (PLC); and waist-hip ratio (WHR). Here, we list the number of significant genes found when using gene-ε with various regularization strategies, as well as the number of dbGAP categories enriched for significant genes identified by gene-ε. We also assess how well these results overlap with the gene-ε -EN findings that were reported in the main text. Significant genes were determined by using a Bonferroni-corrected P-value threshold (in our analyses, P = 0.05/17680 autosomal genes = 2.83×10^−6^). Enriched dbGAP categories were those with Enrichr Q-values (i.e., false discovery rates) less than 0.05. (PDF) [file pgen.1008855.s056.pdf]

	Trait	OLS	Ridge Regression	LASSO	Elastic Net
# Sig. Genes	Height	859	21	90	71
	BMI	770	9	14	73
	MCV	564	90	86	104
	MPV	595	119	83	80
	PLC	517	73	75	69
	WHR	721	4	25	4
% Sig. Gene Overlap w/ Elastic Net	Height	6.82%	42.86%	70.00%	—
	BMI	1.69%	11.11%	64.29%	—
	MCV	13.83%	46.67%	83.72%	—
	MPV	12.77%	50.42%	84.34%	—
	PLC	11.99%	42.47%	85.33%	—
	WHR	0.28%	0.00%	12.00%	—
# Enriched dbGaP Categories	Height	3	1	1	1
	BMI	30	7	0	0
	MCV	2	2	4	1
	MPV	9	4	2	3
	PLC	5	1	1	1
	WHR	10	0	0	0
% Enriched dbGaP Overlap w/ Elastic Net	Height	33.33% (Body Height)	100.00% (Body Height)	100.00% (Body Height)	—
	BMI	0.00%	0.00%	0.00%	—
	MCV	50.00% (Erythrocyte Indices)	50.00% (Erythrocyte Indices)	25.00% (Erythrocyte Indices)	—
	MPV	11.11% (Platelet Count)	75.00% (Platelet Count; Hearing Loss; Face)	100.00% (Platelet Count; Face)	—
	PLC	20.00% (Platelet Count)	100.00% (Platelet Count)	100.00% (Platelet Count)	—
	WHR	0.00%	0.00%	0.00%	—
