# Supplementary material for: Estimation of non-null SNP effect size distributions enables the detection of enriched genes underlying complex traits
Source: PLoS Genet. 2020 Jun 15;16(6):e1008855. doi: 10.1371/journal.pgen.1008855 (PMC7316356; doi:10.1371/journal.pgen.1008855)
Supplement: S1 Text — Specifically, we give description of data quality control procedures, simulation setup and scenarios, review of other competing gene-level association methods, and additional results for the traits analyzed from the UK Biobank. (PDF) [file pgen.1008855.s057.pdf]

Supporting Information to "Estimation of Non-null SNP Effect Size Distributions Enables the Detection of Enriched Genes Underlying Complex Traits"

Wei Cheng[1,2], Sohini Ramachandran[1,2†], and Lorin Crawford[2-4†]

1 Department of Ecology and Evolutionary Biology, Brown University, Providence, RI, USA
2 Center for Computational Molecular Biology, Brown University, Providence, RI, USA
3 Department of Biostatistics, Brown University, Providence, RI, USA
4 Center for Statistical Sciences, Brown University, Providence, RI, USA

† Corresponding E-mail: sramachandran@brown.edu; lorin_crawford@brown.edu

Contents

S1 Data Quality Control Procedures **2**

S2 Simulation Setup and Scenarios **2**

S3 Review of Other Gene-Level Association Methods **3**

S4 Additional Detailed Results for Traits in the UK Biobank **6**

References **7**

S1 Data Quality Control Procedures

The results presented in the main text made use of imputed data released from the UK Biobank [1]. Quality control procedures for these data are as follows. First, we only studied individuals who self-identified as "white British" people. From this cohort, we further excluded individuals identified by the UK Biobank to have high heterozygosity, excessive relatedness, or aneuploidy (1,550 individuals removed). We also removed individuals whose kinship coefficient was greater than 0.0442 (i.e., close relatives). Next, we removed *(i)* monomorphic SNPs, *(ii)* ambiguous A/T or C/G SNPs, *(iii)* SNPs with minor allele frequency (MAF) less than 2.5%, *(iv)* SNPs not in Hardy-Weinberg Equilibrium (Fisher's exact test $P > 10^{-6}$), *(v)* SNPs with missingness greater than 1%, and *(vi)* SNPs in high linkage disequilibrium (using the flag `--indep-pairwise 50 5 0.9` with PLINK 1.9 [2]). After all QC steps, we had a final dataset of 349,414 individuals and 1,070,306 SNPs. Next, we used the NCBI's Reference Sequence (RefSeq) database in the UCSC Genome Browser [3] to annotate SNPs with the appropriate genes. Recall that in both the simulation studies and real data analysis, we define genes with boundaries in two ways: *(a)* we use the UCSC gene boundary definitions directly, or *(b)* we augment the gene boundaries by adding SNPs within a ±50 kilobase (kb) buffer to account for possible regulatory elements. Genes with only 1 SNP within their boundary were excluded from either analysis. A total of 14,322 autosomal genes were analyzed when using the UCSC boundaries, and a total of 17,680 autosomal genes were analyzed when including the 50kb buffer.

S2 Simulation Setup and Scenarios

In our simulation studies, we used the following general simulation scheme to generate SNP-level summary statistics for GWA studies using real genotype data on chromosome 1 from individuals of European ancestry in the UK Biobank [1]. We will denote this genotype matrix as $\mathbf{X}$, with $\mathbf{x}_j$ denoting the genotypic vector for the j-th SNP. Following quality control procedures detailed in the previous section, our simulations included $J = 36{,}518$ SNPs distributed across genome. Again, we used the NCBI's RefSeq database in the UCSC Genome Browser to assign SNPs to genes. Simulations were conducted using two different SNP-to-gene assignments. In the first, we directly used the UCSC annotations which resulted in 1,408 genes to be used in the simulation study. In the second, we augmented the UCSC gene boundaries to include SNPs within ±50kb resulting in 1,916 genes for analysis. Regardless of annotation type, we simulated phenotypes by first assuming that the total phenotypic variance $\mathbb{V}[\mathbf{y}] = 1$ and that all observed genetic effects explained a fixed proportion of this value (i.e., narrow-sense heritability, h^2). Next, we randomly selected a certain percentage of enriched genes and denoted the sets of SNPs that they contained as $\mathcal{C}$. Within $\mathcal{C}$, we select causal SNPs in a way such that each associated gene at least contains one SNP with non-zero effect size. Quantitative continuous traits were then generated under the following two general linear models:

(i) Standard Model: $\mathbf{y} = \sum_{c \in \mathcal{C}} \mathbf{x}_c \beta_c + \mathbf{e}$

(ii) Population Stratification Model: $\mathbf{y} = \mathbf{W}\mathbf{b} + \sum_{c \in \mathcal{C}} \mathbf{x}_c \beta_c + \mathbf{e}$

where $\mathbf{y}$ is an N-dimensional vector containing all the phenotypes; $\mathbf{x}_c$ is the genotype for the c-th causal SNP encoded as 0, 1, or 2 copies of a reference allele; β_c is the additive effect size for the c-th SNP; and $\mathbf{e} \sim \mathcal{N}(0, \tau^2\mathbf{I})$ is an N-dimensional vector of normally distributed environmental noise. Additionally, in model *(ii)*, $\mathbf{W}$ is an $N \times M$ matrix of the top five principal components (PCs) from the genotype matrix and represents additional population structure with corresponding fixed effects $\mathbf{b}$. The effect sizes of SNPs in enriched genes are randomly drawn from standard normal distributions and then rescaled so they explain a fixed proportion of the narrow-sense heritability $\mathbb{V}[\sum \mathbf{x}_c \beta_c] = h^2$. The coefficients for the genotype PCs are also drawn from standard normal distributions and rescaled such that

$\mathbb{V}[\mathbf{Wb}] = 10\%$ of the total phenotypic variance, with the variance of all non-genetic effects contributing $\mathbb{V}[\mathbf{Wb}]+\mathbb{V}[\mathbf{e}] = (1-h^2)$. For any simulations conducted under model (ii), genotype PCs are not included in any of the model fitting procedures, and no other preprocessing normalizations were carried out to account for the additional population structure. More specifically, GWA summary statistics are then computed by fitting a single-SNP univariate linear model via ordinary least squares (OLS):

$$\widehat{\beta}_j = (\mathbf{x}_j^{\intercal}\mathbf{x}_j)^{-1}\mathbf{x}_j^{\intercal}\mathbf{y}; \tag{S1}$$

for every SNP in the data $j = 1, \dots J$. These OLS effect size estimates, along with an empirically LD matrix $\mathbf{\Sigma}$ computed directly from the full $N \times J$ genotype matrix $\mathbf{X}$, are given to gene-ε . We also retain standard errors and P-values for the implementation of competing methods (i.e., VEGAS, PEGASUS, RSS, SKAT, and MAGMA). Given the simulation procedure above, we simulate a wide range of scenarios for comparing the performance of gene-level association approaches by varying the following parameters:

- Number of individuals: N = 5,000 and 10,000;
- Narrow-sense heritability: $h^2 = 0.2$ and 0.6;
- Percentage of enriched genes: 1% and 10%;

Furthermore, we set the number of causal SNPs with non-zero effects to be some fixed percentage of all SNPs located within the designated enriched genes. In the setting where we have 1,408 genes with boundaries defined strictly by RefSeq in UCSC Genome Browser, we set this percentage to be 0.125% in the 1% associated gene case, and 3% in the 10% associated gene case. In the setting where we have 1,916 genes with boundaries augmented by the ±50kb buffer, we set this percentage to be 0.125% in the 1% associated gene case, and 8% in the 10% associated gene case. Lastly, for each simulated dataset, we also selected some number of intergenic SNPs (i.e., SNPs not mapped to any gene) to have non-zero effect sizes. This was done to mimic genetic associations in unannotated regulatory elements. Specifically, 5 randomly selected intergenic SNPs were given non-zero contributions to the trait heritability in the 1% enriched genes case, and 30 intergenic SNPs were selected in the 10% enriched genes case.

All performance comparisons are based on 100 different simulated runs for each parameter combination. We computed gene-level P-values for the gene-ε approaches, PEGASUS, VEGAS, SKAT, and MAGMA. For evaluting the performance of RSS, we compute posterior enrichment probabilities. For all approaches, we assessed:

- The power and false discovery rates when identifying enriched genes at a Bonferroni-corrected threshold ($P = 0.05/1,408$ genes $= 3.55 \times 10^{-5}$; $P = 0.05/1,916$ genes $= 2.61 \times 10^{-5}$ if the ±50kb buffer was used) or median probability model (posterior enrichment probability > 0.5) [4];
- The ability to rank true positive (TP) genes over false positives (FP) via receiver operating characteristic (ROC) and precision-recall curves.

All figures and tables show the mean performances (and standard deviations) across all simulated replicates.

S3 Review of Other Gene-Level Association Methods

In this section, we give a comprehensive review of the three gene-level association tests that we compare with the gene-ε approach. To facilitate the understanding of these summaries, we adapt notation from the original references that first introduced these methods to mirror the notation we use in this study.

Precise, Efficient Gene Association Score Using SNPs (PEGASUS). Consider a gene g with $|\mathcal{J}_g|$ SNPs, where $|\mathcal{J}_g|$ represents the cardinality of the set of SNPs $\mathcal{J}_g$. Also assume that we have access to corresponding $|\mathcal{J}_g|$ GWA SNP-level P-values. We denote the P-values for SNPs within a given gene boundary as $\widehat{\mathbf{p}}_g = \{\widehat{p}_1, \ldots, \widehat{p}_{|\mathcal{J}_g|}\}$. PEGASUS computes a gene-level test statistic $\widehat{Q}_g$ via the following quadratic form

$$\widehat{Q}_g = \widehat{\boldsymbol{\beta}}_g^{\intercal} \mathbf{A} \widehat{\boldsymbol{\beta}}_g \tag{S2}$$

where $\widehat{\boldsymbol{\beta}}_g = F^{-1}(\widehat{\mathbf{p}}_g)$, and $F^{-1}(\bullet)$ is the quantile function of the standard chi-square distribution with one degree of freedom, and $\mathbf{A}$ is a predefined symmetric and positive semi-definite weight matrix. Probabilistically, under the null hypothesis, $\widehat{\boldsymbol{\beta}}_g$ is assumed to jointly follow a multivariate normal distribution with mean $\mathbf{0}$ and covariance matrix $\boldsymbol{\Sigma}_g$, where each matrix element $\rho(\mathbf{x}_j, \mathbf{x}_l)$ is the LD between the j-th and l-th SNPs contained within gene g. Therefore, also under the null hypothesis, Q_g is assumed to follow a mixture of chi-square distributions,

$$Q_g \sim \sum_{j=1}^{|\mathcal{J}_g|} \lambda_j U_j^2 \tag{S3}$$

where each U_j is a mutually independent standard normal variables, and $(\lambda_1, \ldots, \lambda_{|\mathcal{J}_g|})$ are the eigenvalues of the matrix product $\boldsymbol{\Sigma}_g \mathbf{A}$. P-values are computed numerically using Davies' exact method [5]. See [6] for more details. Note that in our implementation of PEGASUS, $\mathbf{A} = \mathbf{I}$ is set to be the identity matrix.

Versatile Gene-based Association Study (VEGAS). Again consider a gene g with $|\mathcal{J}_g|$ SNPs. Under the null hypothesis, a non-associated gene will contain only non-causal SNPs and is assumed to be represented by a $|\mathcal{J}_g|$-dimensional multivariate normal vector $\boldsymbol{\beta}_g^* = (\beta_1^*, \ldots, \beta_{|\mathcal{J}_g|}^*)$ for which

$$\boldsymbol{\beta}_g^* \sim \mathcal{N}(\mathbf{0}, \boldsymbol{\Sigma}_g), \tag{S4}$$

where $\boldsymbol{\Sigma}_g$ is the LD matrix for all SNPs within gene g. VEGAS generates gene scores by: *(i)* simulating the random vector $\boldsymbol{\beta}_g^*$ upwards of one million times, *(ii)* transforming the elements of each vector into correlated chi-square variables with one degree of freedom where $q_j = \beta_j^{*2}$ and $\mathbf{Q}_g^* = (q_1, \ldots, q_{|\mathcal{J}_g|})$, *(iii)* acquiring realizations from the null distribution by summing over all the components in each $\mathbf{Q}_g^*$, and *(iv)* computing an empirical gene-level P-value based on the proportion of times an observed test statistic is smaller than the simulated null statistics $\Pr[\sum \widehat{\mathbf{Q}}_g < \sum \mathbf{Q}_g^*]$ across all simulations. See [7] for more details.

Regression with Summary Statistics (RSS) Enrichment. Consider a GWA study with N individuals typed on P SNPs. For the j-th SNP, assume that we are given corresponding effect sizes $\widehat{\beta}_j$ and standard error $\widehat{s}_j$ via a single-SNP linear model fit using OLS. RSS then implements the following likelihood to model the GWA summary statistics [8]

$$\widehat{\boldsymbol{\beta}} \sim \mathcal{N}(\widehat{\mathbf{S}} \boldsymbol{\Sigma} \widehat{\mathbf{S}}^{-1} \boldsymbol{\beta}, \widehat{\mathbf{S}} \boldsymbol{\Sigma} \widehat{\mathbf{S}}) \tag{S5}$$

where $\widehat{\mathbf{S}} = \text{diag}(\widehat{\mathbf{s}})$ is a $J \times J$ diagonal matrix of standard errors, $\boldsymbol{\Sigma}$ is again used to represent some empirical estimate of the LD matrix (i.e., using some external reference panel with ancestry matching the cohort of interest), and $\boldsymbol{\beta}$ are the true (unobserved) SNP-level effect sizes. To model gene-level enrichment, RSS assumes the following hierarchical prior structure on the true effect sizes

$$\beta_j \sim \pi_j \, \mathcal{N}(0, \sigma_\beta^2) + (1 - \pi_j) \, \delta_0, \tag{S6}$$

$$\sigma_\beta^2 = h^2 \left(\sum_{j=1}^{J} \pi_j \, N^{-1} \widehat{s}_j^{-2} \right)^{-1}, \tag{S7}$$

$$\pi_j = \left(1 + 10^{-(\theta_0 + a_j \theta)}\right)^{-1}, \tag{S8}$$

where δ_0 is point mass centered at zero, h^2 denotes the narrow-sense heritability of the trait, a_j is an indicator detailing whether the j-th SNP is inside a particular gene, θ_0 is the background proportion of trait-associated SNPs, and θ reflects the increase in probability (on the $\log_{10}$-odds scale) when a SNP within a gene has non-zero effect. Here, the authors follow earlier works [9] and place independent uniform grid priors on the hyper-parameters $\{h^2, \theta_0, \theta\}$. Note that, unlike other methods, RSS does not calculate a P-value for assessing gene-level association. Instead, RSS produces a posterior enrichment probability that at least one SNP in a given gene boundary is associated with the trait

$$P_g := 1 - \Pr\left[\beta_j = 0, \forall j \in \mathcal{J}_g \,|\, \mathbf{D}\right] \tag{S9}$$

where $\mathbf{D}$ represents all of the input data including the GWA summary statistics $\{\widehat{\boldsymbol{\beta}}, \widehat{\mathbf{s}}\}$, the estimated LD matrix $\boldsymbol{\Sigma}$, and any applicable SNP annotations or weights $\mathbf{a} = (a_1, \ldots, a_J)$. See [8, 10] for more details on preferred hyper-parameter settings. As noted in the main text, RSS is relies on a Markov chain Monte Carlo (MCMC) scheme for sampling posterior distributions and estimating model parameters. As a result, its algorithm can be subject to convergence issues if these (or the random seed) are not chosen properly.

SNP-set (Sequence) Kernel Association Test (SKAT). The implementation of SKAT required access to raw phenotype $\mathbf{y}$ and genotype $\mathbf{X}$ information for N individuals typed on J SNPs. To assess enrichment of the $|\mathcal{J}_g|$ variants within gene g, consider the linear model with sub-matrix $\mathbf{X}_g$

$$\mathbf{y} = \beta_0 + \mathbf{X}_g \boldsymbol{\beta}_g + \mathbf{e}, \qquad \mathbf{e} \sim \mathcal{N}(\mathbf{0}, \tau^2 \mathbf{I}) \tag{S10}$$

where β_0 is an intercept term, $\boldsymbol{\beta}_g = (\beta_1, \ldots, \beta_{|\mathcal{J}_g|})$ is a vector of regression coefficients for the SNPs within the gene of interest, and $\mathbf{e}$ is a normally distributed error term with mean zero and scaled variance τ^2. For model flexibility, gene-specific SNP effects β_j are assumed to follow an arbitrary distribution with mean zero and marginal variances $a_j \sigma_\beta^2$, where σ_β^2 is a variance component and a_j is a pre-specified weight for the j-th SNP. To this end, SKAT uses a variance component scoring approach and tests the null hypothesis $H_0 : \boldsymbol{\beta} = \mathbf{0}$, or equivalently $H_0 : \sigma_\beta^2 = 0$. The corresponding gene-level test statistic $\widehat{Q}_g$ then takes on the familiar quadratic form

$$\widehat{Q}_g = (\mathbf{y} - \widehat{\beta}_0)^\intercal \mathbf{K}_g (\mathbf{y} - \widehat{\beta}_0) \tag{S11}$$

where $\widehat{\beta}_0$ is the predicted mean of trait under the null hypothesis, and is computed by projecting $\mathbf{y}$ onto the column space of the intercept (i.e., a vector of ones). The term $\mathbf{K}_g = \mathbf{X}_g \mathbf{A}_g \mathbf{A}_g \mathbf{X}_g^\intercal$ is commonly referred to as an $N \times N$ kernel matrix, where $\mathbf{A}_g = \mathrm{diag}(a_1, \ldots, a_{|\mathcal{J}_g|})$ is used to denote a diagonal weight matrix that changes for each gene g. Each element of $\mathbf{K}_g$ is computed via the linear kernel function

$$k(\mathbf{x}_i, \mathbf{x}_{i'}) = \sum_{j=1}^{|\mathcal{J}_g|} a_j x_{ij} x_{i'j}. \tag{S12}$$

While implementing SKAT in this work, we follow previous works and set each weight to be $\sqrt{a_j} = \mathrm{Beta}(\mathrm{MAF}_j, 1, 25)$ — the beta distribution density function with pre-specified parameters evaluated at the sample minor allele frequency (MAF) for the j-th SNP in the gene region. For more details, see [11–14].

Multi-marker Analysis of GenoMic Annotation (MAGMA). In the current study, gene analyses with MAGMA also required access to raw phenotype $\mathbf{y}$ and genotype $\mathbf{X}$ information for N individuals typed on J SNPs. This approach is based on a multiple principal components regression model. In the first step, MAGMA projects the sub-genotype matrix for a gene $\mathbf{X}_g$ onto its principal components. Next, it prunes away PCs with very small eigenvalues, and then uses those reduced vectors as predictors for the phenotype in the linear regression model. Consider the following linear regression and singular-value decomposition (SVD) of the genotype matrix

$$\mathbf{y} = \beta_0 + \mathbf{X}\boldsymbol{\beta} + \mathbf{e}, \qquad \mathbf{X} = \mathbf{U}\boldsymbol{\Lambda}\mathbf{V}^\intercal, \qquad \mathbf{e} \sim \mathcal{N}(\mathbf{0}, \tau^2\mathbf{I}) \tag{S13}$$

where, in addition to the aforementioned notation, $\boldsymbol{\Lambda}$ is an $N \times J$ rectangular diagonal matrix of singular values, and $\mathbf{U}$ and $\mathbf{V}$ are $N \times N$ and $J \times J$ matrices of orthogonal unit vectors, respectively. For numerical stability and reduction of computational complexity, vectors corresponding to small eigenvalues can be truncated. Therefore, without loss of generality, MAGMA considers $\mathbf{V}$ and $\boldsymbol{\Lambda}$ to be of dimensions $J^* \times J^*$ and $N \times J^*$, respectively. Here, J^* denotes the top eigenvalues explaining 99.9% of the cumulative variance in $\mathbf{X}_g$. By defining $\mathbf{G} = \mathbf{U}\boldsymbol{\Lambda}$, the model above simplifies to

$$\mathbf{y} = \beta_0 + \mathbf{G}\boldsymbol{\vartheta} + \mathbf{e}, \qquad \mathbf{e} \sim \mathcal{N}(\mathbf{0}, \tau^2\mathbf{I}) \tag{S14}$$

where $\boldsymbol{\vartheta} = \mathbf{V}^\intercal\boldsymbol{\beta}$ represents the lower-dimensional genetic effect. To derive a P-value for a single gene's association with the phenotype, MAGMA uses an F-test under the null hypothesis $H_0 : \boldsymbol{\vartheta} = \mathbf{0}$ or, equivalently, $H_0 : \mathbf{V}^\intercal\boldsymbol{\beta} = \mathbf{0}$. See [15] for more details.

S4 Additional Detailed Results for Traits in the UK Biobank

In this section, we present additional detailed findings and results from applying gene-ε to the six quantitative traits — height, body mass index (BMI), mean red blood cell volume (MCV), mean platelet volume (MPV), platelet count (PLC), waist-hip ratio (WHR) — assayed in self-identified European-ancestry individuals in the UK Biobank [1]. For these extra set of analyses, we obtained the genotype data release (without imputed genotypes) and implemented the same quality control procedure that was used in the main text (Section S1). This resulted in a final dataset of N = 349,468 individuals and J = 410,172 genome-wide SNPs. Once again, we used the NCBI's Reference Sequence (RefSeq) database in the UCSC Genome Browser [3] to annotate SNPs with the appropriate genes in one of two ways. In the first setting, we use the UCSC gene boundary definitions directly; while in the second setting, we augment the gene boundaries by adding SNPs within a ±50 kilobase (kb) buffer to account for possible regulatory elements. Genes with only 1 SNP in their boundary were excluded from the respective analysis. For these data, a total of 13,029 autosomal genes were analyzed when using the UCSC boundaries as defined; while, a total of 17,680 autosomal genes were analyzed when including the 50kb buffer. Lastly, we regressed the top ten principal components of the genotype data onto each trait to control for population structure, and then we derived OLS SNP-level effect sizes using the traditional GWA framework. Here, our goal is to compare how the four different implementations of gene-ε (i.e., OLS with no regularization, Ridge Regression, Elastic Net, and LASSO) analyze these summary statistics.

As shown in the main text, we begin with assessing how the various regularization solutions result in different characterizations of genetic architectures (S25 Table). In general, we find the same general themes we saw in our simulation study. Less aggressive shrinkage approaches (e.g., OLS and Ridge) are subject to misclassifications of associated, spurious, and non-associated SNPs. As result, these methods struggle to avoid identifying false positive SNP-level associations, across all six traits. For example, gene-ε-OLS assumes that approximately 54% and 50% of the SNPs analyzed are associated with BMI and WHR, respectively. This once again highlights the need for computational frameworks that are able to appropriately correct for inflation in summary statistics.

Lastly, we applied each version of gene-ε to the (regularized) GWA summary statistics and generated genome-wide gene-level association P-values. Recall that we are motivated to identify enriched genes, which we define as a gene containing at least one associated SNP and achieving a gene-level association P-value below a Bonferroni-corrected significance threshold. In our analyses, this significance threshold is $P = 0.05/13029$ autosomal genes $= 3.84 \times 10^{-6}$ when the UCSC gene boundaries are used directly, and $P = 0.05/17680$ autosomal genes $= 2.83 \times 10^{-6}$ when the $\pm$50kb buffer is applied, respectively. As a validation step, we used the gene set enrichment analysis tool Enrichr [16] to identify dbGaP categories with an overrepresentation of significant genes reported by the four different implementations of gene-ε. A comparison of gene-level associations and gene set enrichments between the each gene-ε approaches are also listed (S26 and S27 Tables). Note that, similar to the main text, we use the findings of gene-ε-EN as the reference.

References

1. Bycroft C, Freeman C, Petkova D, Band G, Elliott LT, Sharp K, et al. The UK Biobank resource with deep phenotyping and genomic data. Nature. 2018;562(7726):203–209. Available from: https://doi.org/10.1038/s41586-018-0579-z.

2. Purcell S, Neale B, Todd-Brown K, Thomas L, Ferreira MA, Bender D, et al. PLINK: a tool set for whole-genome association and population-based linkage analyses. Am J Hum Genet. 2007;81(3):559–575.

3. Pruitt KD, Tatusova T, Maglott DR. NCBI Reference Sequence (RefSeq): a curated non-redundant sequence database of genomes, transcripts and proteins. Nucleic Acids Res. 2005;33(Database issue):D501–4.

4. Barbieri MM, Berger JO. Optimal predictive model selection. Ann Statist. 2004;32(3):870–897. Available from: http://projecteuclid.org/euclid.aos/1085408489.

5. Davies RB. Algorithm AS 155: The distribution of a linear combination of χ^2 random variables. J R Stat Soc Ser C Appl Stat. 1980;29(3):323–333. Available from: http://www.jstor.org/stable/2346911.

6. Nakka P, Raphael BJ, Ramachandran S. Gene and network analysis of common variants reveals novel associations in multiple complex diseases. Genetics. 2016;204(2):783–798. Available from: http://www.genetics.org/content/204/2/783.abstract.

7. Liu JZ, Mcrae AF, Nyholt DR, Medland SE, Wray NR, Brown KM, et al. A versatile gene-based test for genome-wide association studies. Am J Hum Genet. 2010;87(1):139–145.

8. Zhu X, Stephens M. Bayesian large-scale multiple regression with summary statistics from genome-wide association studies. Ann Appl Stat. 2017;11(3):1561–1592. Available from: https://projecteuclid.org:443/euclid.aoas/1507168840.

9. Carbonetto P, Stephens M. Integrated enrichment analysis of variants and pathways in genome-wide association studies indicates central role for IL-2 signaling genes in type 1 diabetes, and cytokine signaling genes in Crohn's disease. PLoS Genet. 2013;9(10):e1003770–. Available from: https://doi.org/10.1371/journal.pgen.1003770.

10. Zhu X, Stephens M. Large-scale genome-wide enrichment analyses identify new trait-associated genes and pathways across 31 human phenotypes. Nat Comm. 2018;9(1):4361.

11. Wu MC, Kraft P, Epstein MP, Taylor DM, Chanock SJ, Hunter DJ, et al. Powerful SNP-set analysis for case-control genome-wide association studies. Am J Hum Genet. 2010;86(6):929–942.

12. Wu MC, Lee S, Cai T, Li Y, Boehnke M, Lin X. Rare-variant association testing for sequencing data with the sequence kernel association test. Am J Hum Genet. 2011;89(1):82–93.

13. Lee S, Emond MJ, Bamshad MJ, Barnes KC, Rieder MJ, Nickerson DA, et al. Optimal unified approach for rare-variant association testing with application to small-sample case-control whole-exome sequencing studies. Am J Hum Genet. 2012;91(2):224–237. Available from: `http://www.sciencedirect.com/science/article/pii/S0002929712003163`.

14. Ionita-Laza I, Lee S, Makarov V, Buxbaum JD, Lin X. Sequence kernel association tests for the combined effect of rare and common variants. Am J Hum Genet. 2013;92(6):841–853. Available from: `http://www.sciencedirect.com/science/article/pii/S0002929713001766`.

15. de Leeuw CA, Mooij JM, Heskes T, Posthuma D. MAGMA: generalized gene-set analysis of GWAS data. PLOS Comput Biol. 2015;11(4):e1004219–. Available from: `https://doi.org/10.1371/journal.pcbi.1004219`.

16. Chen EY, Tan CM, Kou Y, Duan Q, Wang Z, Meirelles GV, et al. Enrichr: interactive and collaborative HTML5 gene list enrichment analysis tool. BMC Bioinform. 2013;14(1):128. Available from: `https://doi.org/10.1186/1471-2105-14-128`.
