# Supplementary material for: Estimation of non-null SNP effect size distributions enables the detection of enriched genes underlying complex traits
Source: PLoS Genet. 2020 Jun 15;16(6):e1008855. doi: 10.1371/journal.pgen.1008855 (PMC7316356; doi:10.1371/journal.pgen.1008855)
Supplement: S26 Fig — BMI has been estimated to have a narrow-sense heritability h2 ranging from 0.25 to 0.60 [31, 33, 34, 36, 37, 39, 42–45]. Manhattan plots of gene-ε gene-level association P-values using Elastic Net regularized effect sizes when gene boundaries are defined by (A) using UCSC annotations directly, and (B) augmenting the gene boundaries by adding SNPs within a ±50kb buffer. The purple dashed line indicates a log-transformed Bonferroni-corrected significance threshold (P = 3.49×10^−6^ and P = 2.83×10^−6^ correcting for the 14,322 and 17,680 autosomal genes analyzed, respectively). We color code all significant genes identified by gene-ε in orange, and annotate genes previously associated with BMI in the database of Genotypes and Phenotypes (dbGaP). In (C) and (D), we conduct gene set enrichment analysis using Enrichr [46, 59] to identify dbGaP categories enriched for significant gene-level associations reported by gene-ε in (A) and (B), respectively. While many of the scored categories are biologically related to BMI (e.g., “Body Mass Index”, “Adiposity”, and “Arteries”) [66, 114–116], none of them had Q-values (i.e., false discovery rates) less than 0.05. (PDF) [file pgen.1008855.s026.pdf]

A

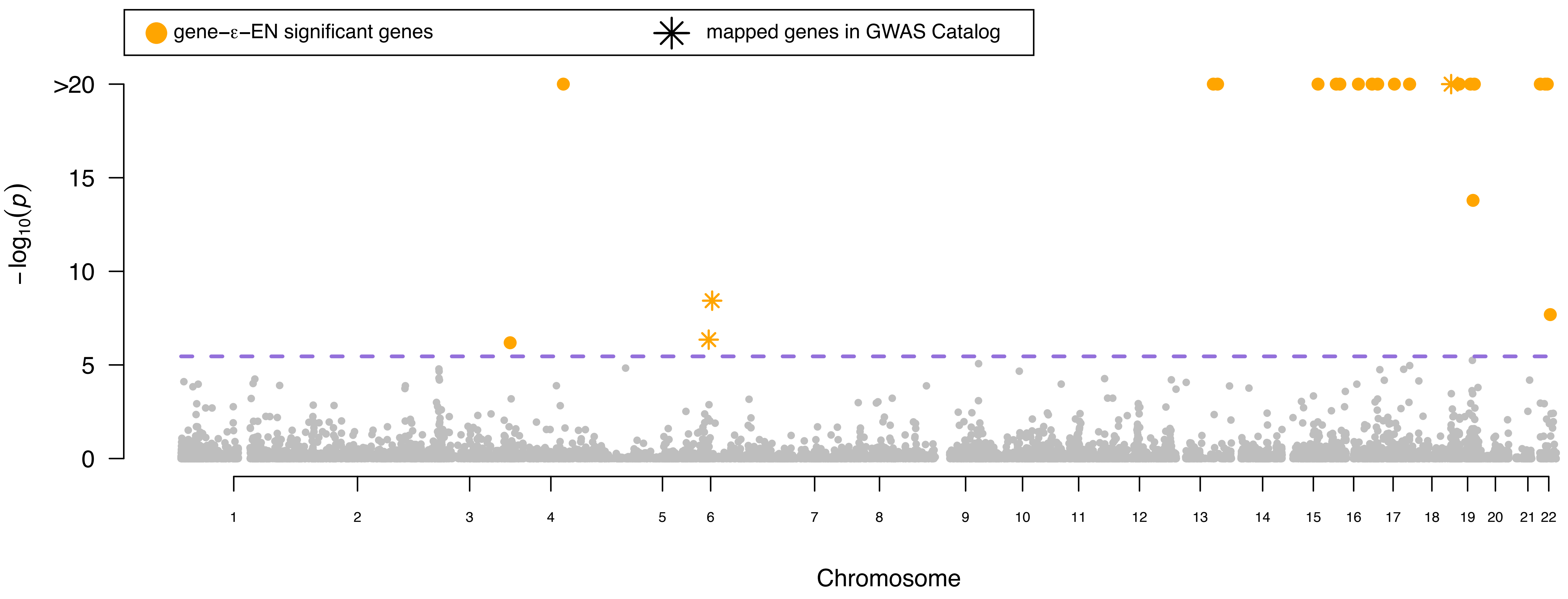

B

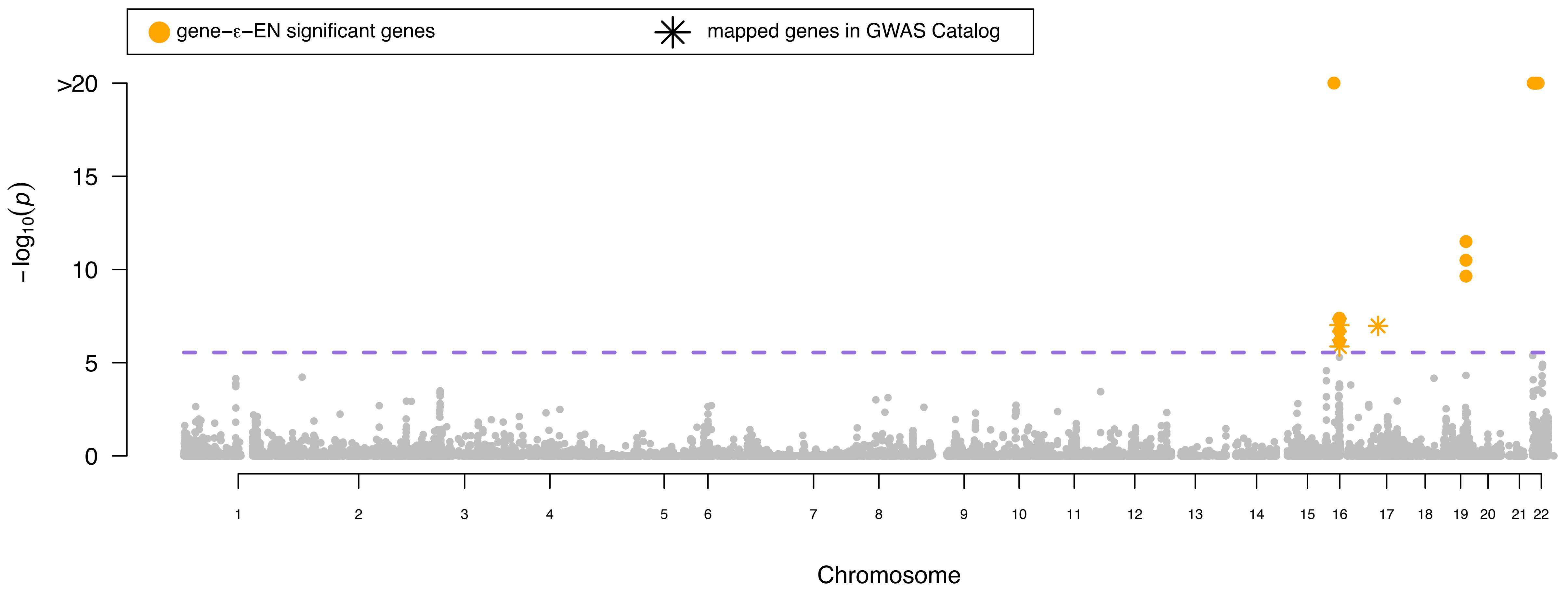

C

	p value	*q* value	Odds.ratio	Combined score	# of sig. genes in dbGaP
Adiposity	4.79e-03	1.00	208.33	1112.68	1
Metabolic Syndrome X	2.73e-02	1.00	36.23	130.53	1
Body Mass Index	9.59e-02	1.00	3.81	8.94	2
—	—	—	—	—	—
—	—	—	—	—	—
—	—	—	—	—	—
—	—	—	—	—	—
—	—	—	—	—	—
—	—	—	—	—	—
—	—	—	—	—	—

D

	p value	*q* value	Odds.ratio	Combined score	# of sig. genes in dbGaP
gamma-Glutamyltransferase	1.85e-02	1.00	53.48	213.24	1
Alkaline Phosphatase	1.94e-02	1.00	51.15	201.72	1
Arteries	7.30e-02	1.00	13.22	34.59	1
Body Mass Index	3.13e-01	1.00	2.69	3.13	1
—	—	—	—	—	—
—	—	—	—	—	—
—	—	—	—	—	—
—	—	—	—	—	—
—	—	—	—	—	—
—	—	—	—	—	—
